# Improved ESI-MS Sensitivity via an Imidazolium Tag (DAPMI-ITag) for Precise Sialic Acid Detection in Human Serum and CMAH-Null Mouse Tissues

**DOI:** 10.1021/acs.analchem.5c00752

**Published:** 2025-06-09

**Authors:** Yao-Yao Zhang, Zi-Xuan Hu, Si-Yu Zhang, Li Liu, M. Carmen Galan, Josef Voglmeir, Mattia Ghirardello

**Affiliations:** † Glycomics and Glycan Bioengineering Research Center (GGBRC), College of Food Science and Technology, 70578 Nanjing Agricultural University, 1 Weigang, 210095 Nanjing, China; ‡ Lipid Technology and Engineering, School of Food Science and Engineering, Henan University of Technology, Lianhua Road 100, 450001 Zhengzhou, China; § School of Chemistry, University of Bristol, Cantock’s Close, BS8 1TS Bristol, U.K.; ∥ Institute of Biocomputation and Physics of Complex Systems (BIFI), University of Zaragoza, Calle Mariano Esquillor, Edificio I+D, 50018 Zaragoza, Spain

## Abstract

Sialic acids (Sias), consisting primarily of *N*-acetylneuraminic acid (Neu5Ac) and *N*-glycolylneuraminic acid (Neu5Gc), play crucial roles in many biological processes. The detection and quantification of Sias are essential for understanding their roles in health and disease progression. Although numerous techniques have been developed to enhance the specificity and sensitivity of Sias analysis, traditional methods such as derivatization with fluorescent tags coupled with HPLC-MS analysis often suffer from low limits of detection, limiting the quantification of Sias in trace samples. Here, we introduce DAPMI, a novel imidazolium-based ITag for sensitive Sia detection. We demonstrate its utility in the detection and quantification of Sia composition in human serum, and in different tissues from CMAH (cytidine monophosphate-*N*-acetylneuraminic acid hydroxylase) knockout mice, using ESI-MS analysis and with a limit of detection (LOD) down to the low fmol range. The results showed that both Neu5Ac and Neu5Gc were present in varying proportions in wild-type mice and CMAH heterogeneous mice. Trace amounts of Neu5Gc were also detected in the tissues of CMAH null homogeneous mice (CMAH–/−) and in human blood serum using ESI-ToF-MS, suggesting its presence may be linked to dietary intake of Neu5Gc-containing foods, as Neu5Gc cannot be synthesized endogenously in CMAH–/– mice, and in humans. The DAPMI-ITag and the labeling technology developed in this study significantly improve the sensitivity of Sias detection compared to conventional tags such as *o*-phenylenediamine (OPD), and provide a new chemical tool for the exploration of Sias’ biological roles and their use as biomarkers in different human conditions.

## Graphical abstract

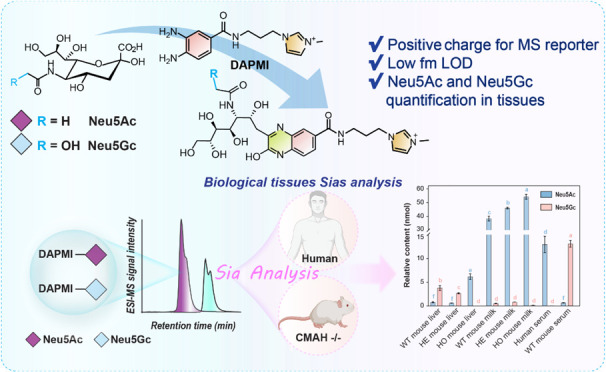

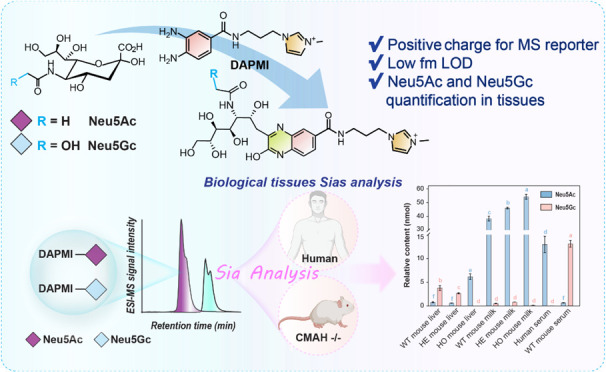

## Introduction

Sialic acids (Sias) are charged monosaccharides with a nine-carbon backbone and are generally present at the terminal region of glycan chains decorating glycoproteins and glycolipids. The predominant forms of Sias found in mammals are *N*-acetylneuraminic acid (Neu5Ac) and *N*-glycolylneuraminic acid (Neu5Gc).^1,2^ Sias are mainly present as terminal residues in α-2,3 or α-2,6 *O*-glycans,^3^ and are ubiquitously expressed in different tissues such as neural tissues, epithelial cells, and red blood cells.^4,5^ Sialoglycans play pivotal roles in numerous biological processes, including mediating cell–cell interactions,^6^ facilitating cell signaling,^7,8^ and supporting the proper functioning of the nervous system.^1,9^

Sias are also implicated in the progression of human diseases, serving as primary binding sites for pathogens such as viruses, which use Sias on the cell surface as anchoring points during the initial phase of the host cell infection process.^10–12^ Additionally, aberrant sialylation has been implicated in the pathogenesis of cardiovascular disease, diabetes, cancer, and inflammatory and degenerative disorders.^13,14^ In the case of cancer, Sias overexpression has been shown to promote the formation of an immunosuppressive microenvironment favoring cancer growth and the development of metastasis.^15–19^ For instance, overexpression of sialylated glycans, particularly α-2,6-sialyl glycosides, was observed in mid- to late-stage colorectal cancer compared to early-stage tumors, and was also identified as the predominant Sia glycoform in colorectal cancer cells.^20^ Therefore, the global Sias level can serve as a prognostic and therapeutic biomarker, facilitating the early diagnosis of colorectal and different types of cancer.

In humans, Neu5Gc is absent due to the inactivation of cytidine monophosphate-*N*-acetylneuraminic acid hydroxylase (CMAH) by a 92 bp exon deletion that blocks the addition of a hydroxyl group in CMP-Neu5Ac to generate CMP-Neu5Gc.^21,22^ Comparative genomics indicates that chimpanzees, gorillas, baboons, and rhesus monkeys harbor a primordial AluSq retroposon sequence within their genomes, a sequence that is notably absent in the human DNA segment encoding the CMAH (Figure 1A).^23^ This genetic difference arose as an adaptation mechanism in human ancestors, reducing their susceptibility to Neu5Gc-binding pathogens such as the malaria parasite.^24^ However, substantial evidence confirms the presence of nonhuman Neu5Gc and its corresponding antibodies in humans, which correlates with the consumption of dairy products and red meat. Despite being an exogenous antigen, trace amounts of Neu5Gc can be metabolically incorporated into human glycoproteins from animal-derived dietary sources. The interaction with circulating anti-Neu5Gc antibodies leads to the development of inflammation, which has been identified as a potential contributor to cancer development.^25^ Therefore, Neu5Gc can serve as a biomarker for the detection of poor dietary habits that can place individuals at higher risks of contracting cancer.^26^

**Figure 1:**
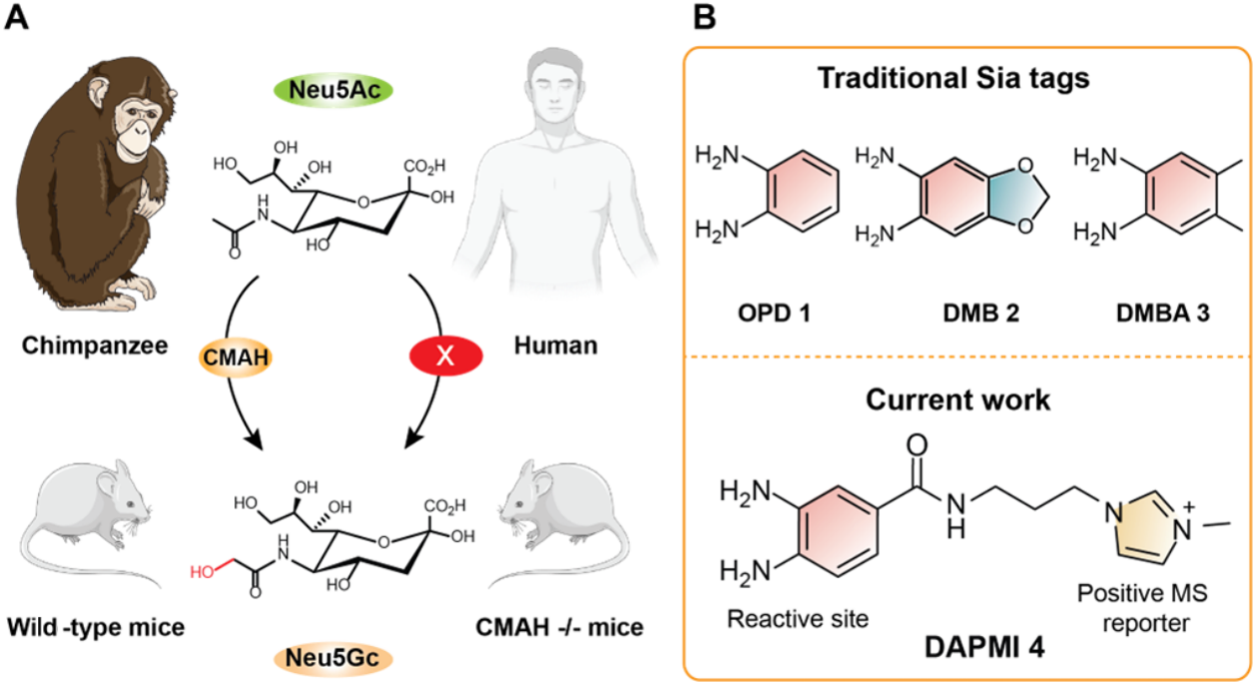
(A) Structure of Neu5Ac and conversion into Neu5Gc in chimpanzee and wild-type mice. (B) Structures of traditional Sia tags **1**-**3** and novel DAPMI tag **4**.

Given the pivotal role of Sias in biological and physiological processes, precise, facile, and sensitive quantification of specific Sia species and their total content is key for early disease diagnoses and a comprehensive assessment of human health statuses.^27–29^

It is noteworthy that the Sia residues in their bound forms are mostly found in the terminal part of glycoconjugates. Consequently, to achieve an accurate quantification of Sias, it is essential to release them from their bound state through acid hydrolysis of the glycosidic bond. This facilitates subsequent derivatization steps required to enhance Sias detection sensitivity. Various techniques have been developed for the quantification of Sias and their derivatives including fluorometric, high-performance liquid chromatography (HPLC), colorimetric and mass spectrometry (MS) assays, among others.^30–34^ However, the structural lability of Sia glycosidic linkages and the inherent negative charge of these moieties make them challenging analytes for MS detection in positive ion mode, hampering the accurate identification and relative Sias quantification.^29^ Additionally, the formation of heterogeneous adducts with different counterions such as hydrogen, sodium, potassium, and ammonium ions reduces the ionization efficiency and the mass accuracy, complicating the Sias analysis in negative ion mode.^30^ Although the derivatization of the carboxylic group of Sias into methyl esters and amide derivatives has proven effective for improving mass spectrometry (MS) detection sensitivity, considerable improvements are still needed to optimize ionization efficiency.^29^

Currently, *o*-phenylenediamine (OPD, **1**), 1,2-diamino-4,5-methylenedioxybenzene dihydrochloride (DMB, **2**), and 4,5-dimethylbenzene-1,2-diamine (DMBA, **3**) are among the most common fluorescent tags used for Sia derivatization,^35–37^ helping to facilitate detection efficiency and prevent Sia loss during sample treatment (Figure 1B). However, the poor separation efficiency of Sias derivatized with DMB in HPLC and the suboptimal ionization sensitivity of OPD and DMBA-labeled Sias in MS detection have motivated us to search for an alternative Sia derivatization strategy with higher ionization efficiency and sensitivity to detect trace analytes in complex biological samples.

Previous studies demonstrated how the presence of a permanent positive charge in glycan labels such as Girard’s reagents^38^ and the QUANTITY tag^39^ is an effective strategy to enhance the MS detection sensitivity. We also demonstrated that quaternary imidazolium salts (GI-Tags) bearing a permanent positive charge significantly enhance ionization efficiency in MS analyses for neutral *N*-glycans.^40,41^ These imidazolium-based labels enabled the detection of trace levels of *N*-glycans using ESI-MS techniques, consistently eliminating additional ionic adducts (e.g., H^+^, Na^+^, K^+^). However, the labels were less effective for the detection of charged species, and sialylated species were detected less efficiently. We thus propose that combining the high MS sensitivity of an imidazolium tag with the fluorescent features of *o*-phenylenediamine derivatizing agents, such as compounds **1–3**, could significantly improve detection efficiency. We hypothesize that a reagent such as diamino phenyl 1-(3-(3,4-diaminobenzamido)­propyl)-3-methyl-1*H*-imidazol-3-ium (DAPMI) **4** could selectively react with the acidic moieties in Sias, while concomitantly converting them into positively charged species for enhanced MS sensitivity (Figure 1B and Scheme 1B).

**Scheme 1:**
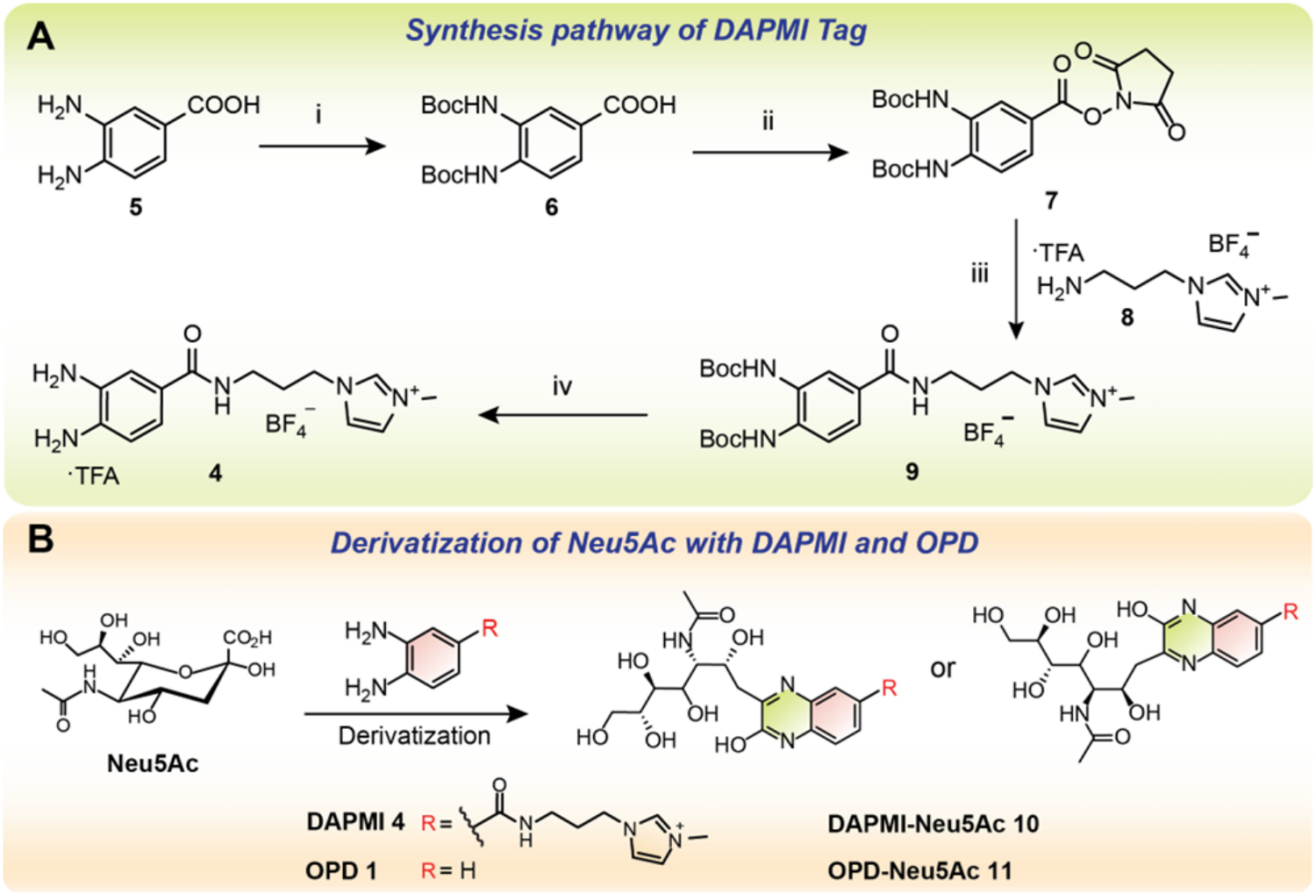
(A) Synthetic Route for the Synthesis of the Sia Tag of DAMPI^a^ and (B) Derivatization Procedure of Neu5Ac with DAMPI **4** and OPD **1** ^b^

a Reagents and conditions: (i) Boc_2_O, TEA, H_2_O/1,4-dioxane, 16 h, r.t., 29%; (ii) NHS, DCC, DMF, 16 h, r.t., 92%; (iii) **8**, DIPEA, DMF, 16 h, r.t., 92%; (iv) TFA, DCM, 2 h, r.t., quant.
b Derivatization conditions: NaHSO_3_, 80 °C, 45 min.

Initial tests to assess the tagging efficiency and detection sensitivity of DAPMI used Neu5Ac as a model Sia. Following these preliminary evaluations, DAPMI’s tagging efficiency was further validated in several biological samples including human serum and CMAH null (CMAH–/−) mouse serum, milk, and liver samples. This animal model was chosen for its phenotypic similarity to humans and as a commonly used system to study the disorders caused by the evolutionary loss of Neu5Gc in humans.^42^ HPLC-ESI-MS analysis demonstrated that DAMPI is a highly efficient and sensitive ITag, offering improved detection and quantification accuracy for Sias in complex biological matrices. The DAPMI tag provides an unprecedented detection of Sias using orthogonal analytical techniques. A robust synthetic procedure has been developed to provide a probe that combines a fluorogenic moiety and a cationic imidazolium reporter on the same scaffold, enabling the dual-mode detection of free Sias through both fluorescence and MS analysis, enhancing Sias detection accuracy.

## Materials and Methods

### Imidazolium Tag Preparation and Characterization

3-Bromopropylamine hydrobromide, *o*-phenylenediamine (OPD), 3,4-diaminobenzoic acid, di-*tert*-butyl decarbonate, *N*-methylimidazolium, potassium tetrafluoroborate (KBF_4_), *N*-hydroxysuccinimide (NHS), and *N*,*N*-diisopropylethylamine (DIPEA) were purchased from J&G Chemicals (Nanjing, China). Dichloromethane (CH_2_Cl_2_), ethyl acetate (EtOAc), methanol (MeOH), and acetonitrile (ACN) were obtained from General Reagent Co. (Shanghai, China). ACN used for HPLC analysis was purchased from Merck. (Nanjing, China). Wide-type mice were provided by the Comparative Medicine Centre of Yangzhou University (China). CMAH knockout mice were obtained commercially from Shanghai Model Organisms Center, Inc. (Shanghai, China), generated using a CRISPR/Cas9 strategy to remove 92 base pairs from exon 6 of the CMAH gene.^42,43^ Liver and milk samples were collected from both wild-type and CMAH knockout mice. Human serum was obtained under sterilized conditions from Prof. Josef Voglmeir. Mouse serum (wild-type) was obtained from Yuanye Bio-Technology Co., Ltd. (Shanghai, China). Other chemicals were obtained from commercial suppliers without further treatment. Procedures involving animal subjects have been approved by the Ethical Committee of the Experimental Animal Center of Nanjing Agricultural University, in accordance with the National Guidelines for Experimental Animal Welfare (Ministry of Science and Technology, P. R. China, 2006), and animals were housed in a specific-pathogen-free (SPF) facility (Permission ID: SYXK-J-2011-0037). The preparative-scale synthesis of DAPMI tag **4** was carried out as described in SI Section 1.

### Preparative-Scale Synthesis, Purification, and Characterization of DAPMI- and OPD-Derivatized Neu5Ac

The preparation of the DAPMI-Neu5Ac and the OPD-Neu5Ac conjugates was performed as described in SI Section 1 (Scheme 1). The purified fractions were pooled, lyophilized, and weighed for analysis. The analyses were characterized by Spark Fluorescence Scanner (Thermo Fisher, Inc.) and Shimadzu LCMS 8040 system (Shimadzu Corporation, Kyoto, Japan), consisting of an LC-30AD pump equipped with a low-pressure gradient mixing unit, an SIL-30AC autosampler, and an RF-20Axs fluorescence detector (FLD).

### Optimization of Derivatization Process

Optimization of derivatization conditions, including derivatization time, derivatization temperature, DAPMI concentration, sodium bisulfite (NaHSO_3_) concentration, and derivatization solvent, was performed based on a 20 μL reaction volume composed of 2 μL of Neu5Ac (50 mM), 8 μL of sodium bisulfite (500 mM), and 10 μL of DAPMI (20 mg/mL) aqueous solutions. The derivatization was carried out at 80 °C for 45 min. For derivatization optimization based on time and temperature, reactions were sampled at different time points (15, 30, 45, 60, 75, 90, 105, and 120 min) and at different reaction temperatures (30, 40, 50, 60, 70, 80, 90 and, 100 °C). Further optimization of DAPMI and sodium bisulfite concentration, the Sia derivatization reaction was carried out with different DAPMI concentrations (0.1, 0.2, 0.5, 1, 2, 5, 10, 20, and 50 mg/mL) and NaHSO_3_ concentrations (0, 20, 50, 100, 200, 500, 1000, and 2000 mM). Reaction solvent was also evaluated, 20 μL reaction volume was treated with DAPMI (final concentration of 10 mg/mL) in different solvents (H_2_O, DMSO, MeOH, EtOH, CH_3_CN, acetone, and THF). All samples were diluted 50 times before HPLC analysis.

To evaluate the storage stability of DAPMI-derivatized samples, a 20 μL aliquot of the ITag-Sia derivatization reaction mixture was diluted 50-fold, and 100 μL samples of the diluted solution were stored either at 4 °C in the absence of light, or at 20 °C in the presence or absence of ambient light.

### Free Sia Preparation and Purification

The free Sia preparation from mouse samples (liver, milk, and serum) was conducted following previously established procedures with minor modifications.^44^ The thawed mouse liver (50 mg) was homogenized in a 2 mL glassware grinder by adding 1.2 mL of 2 M acetic acid and transferred to a 2 mL Eppendorf tube. Gently thawed 50 μL of milk or serum was pipetted and followed by the addition of 1.2 mL of 2 M acetic acid. All samples were hydrolyzed through thermal treatment of the mixtures at 80 °C for 4 h. Following centrifugation at 4 °C, 12,000*g*, for 15 min, 1 mL of the supernatant was taken and concentrated using a centrifugal evaporator. The residue was resolubilized into 700 μL of water, vortex-mixed, and ultrasonicated at room temperature for 2 h. The solution was centrifuged at 4 °C, 12,000*g* for 15 min, and 600 μL of the supernatant were transferred into the anion-exchange resin columns (200 mg, Dowex 1 × 8 100–200 Cl), preconditionedwith a single pass of 2 mL acetic acid (2 M) and three washes (2 mL each) with ddH_2_O. Following the addition of 2 mL of ddH_2_O, columns were eluted with 1 mL of ammonium acetate (50 mM) and flow-through fractions were collected and spin-dried under vacuum (see Supporting Information, Scheme S1).

### Derivatisation of Sia in Biological Samples

A 20 μL aliquot of DAPMI solution (10 mg/mL in 0.2 M NaHSO_3_) was added to lyophilized Sias derived from biological samples, including human serum, mouse liver, mouse milk, and mouse serum. Derivatization was performed at 80 °C for 45 min, followed by centrifugation treatment (4 °C, 12,000 rpm, 15 min) (see Supporting Information, Section 3).

### UPLC-ESI-MS Analysis

For DAPMI-Neu5Ac identification, 2 μL aliquots were processed using a Shimadzu HPLC-MS system equipped with UV (254 nm), fluorescence (*E*_x_/*E*_m_ = 356/412 nm), and ESI-MS detectors for the analysis of the product. The mobile phase comprised ammonium formate (50 mM, pH 4.5, solvent A) and acetonitrile (solvent B). The solvent B gradient increased from 12 to 20% over 8 min, reaching 95% in 1 min, and held for 2 min at a total flow rate of 0.5 mL/min (SI Table S1). Purification of preparative-scale DAPMI-derivatized Neu5Ac was performed using an HPLC-SPD unit equipped with the preparative column (Cosmosil 5C18 MS-II, 20 mm ID × 250 mm), with a flow rate of 3 mL/min (see Supporting Information, Table S2).

For OPD-Neu5Ac identification, a 2 μL sample was injected into an LCMS-8040 system coupled with a reversed-phase column (Cosmosil 5C18 MS-II 4.6 mm × 250 mm, Nacalai, Inc., Japan). The analytes were identified using HPLC equipped with UV (254 nm), fluorescence (*E*_x_/*E*_m_ = 354/416 nm), and ESI-MS detectors with a mobile phase containing H_2_O (solvent A), acetonitrile (solvent B), and methanol (solvent C) at a flow rate of 0.5 mL/min. The separation followed a linear gradient of 5–25% B and C for 15 min, 25–40% B and C for 5 min, and held at 40% for 4 min (SI Table S3). Mass detection was set in positive ion mode with the *m*/*z* scan range of 100–700 Da, and the target OPD-Neu5Ac fraction was spin-dried and weighed for further characterization (see Supporting Information, Table S4).

UPLC-ESI-MS analysis and quantification of DAPMI-Neu5Ac and OPD-Neu5Ac were performed with a Shimadzu LCMS 8040 system. A 2 μL sample, comprising two compounds, was separated on a reversed-phase HPLC column (Cosmosil 5C18 MS-II 4.6 mm × 250 mm, Nacalai, Inc., Japan) at a constant flow rate of 0.5 mL/min with fluorometric detection (SI Tables S1 and S3) and identified by ESI-ToF-MS (see Supporting Information, Table S4).

The HPLC analysis using the optimized derivatization process was carried out using a C18 reversed-phase column (Cosmosil 5C18 MS-II 4.6 mm × 250 mm, Nacalai, Inc., Japan) and a fluorescent detector (*E*_x_/*E*_m_ = 356/412 nm). The elution phases consisted of solvent A (50 mM ammonium formate, pH 4.5) and solvent B (acetonitrile) followed by the elution procedure reported in the Supporting Information, Table S1.

The determination of DAPMI tag-labeled Sias in biological samples was also conducted using a UPLC-ESI-MS unit. 5 μL of samples were injected and eluted into an Acquity BEH Amide Column (2.1 mm × 150 mm, 1.7 μm, Waters, Ireland) at 60 °C and analyzed by a Shimadzu UPLC-FLD-MS system. The elution solvents comprised solvent A (50 mM ammonium formate, pH 4.5) and solvent B (acetonitrile) with a total flow rate of 0.4 mL/min. The separation gradient was performed as follows: 88% B from 0 to 1.5 min, 88–70% B from 1.5 to 35 min (SI Table S6). The spectra were recorded using an RF-20Axs fluorescence detector (λ_ex_ = 356 nm, λ_em_ = 412 nm) and an 8040 ESI-ToF detector (positive single ion mode).

## Results and Discussion

### Synthesis and Characterization of DAPMI-ITag

The synthesis of DAPMI was accomplished using a convergent strategy that entailed the integration of the fluorophore, which also serves as the Sia trapping reagent, with a cationic MS reporter (Scheme 1). ITag **8**, which bears the positively charged imidazolium moiety, was synthesized in 3 steps with 56% yield as previously described.^41^ Simultaneously, the fluorophore was prepared from commercially available 3,4-diaminobenzoic acid **5**, which was treated with Boc_2_O to give bis-Boc-protected derivative **6** in 29% yield. Subsequent activation of the carboxylic acid via an *N*-hydroxysuccinimide (NHS) ester using DCC as coupling agent, furnished **7** in 92% yield, which upon reaction with amino ITag **8** under basic conditions afforded amide **9** in another 92% yield. Finally, Boc-deprotection of **9** with TFA gave DAPMI **4** in a near-quantitative yield, which was directly used in the labeling procedures without further purification.

### Optimization of Derivatization Process

To optimize the DAPMI labeling efficiency, a Neu5Ac 5 mM aqueous solution was chosen as a standard derivatizing model and HPLC integration of the product peak was used as the key monitoring parameter. Different labeling conditions were screened, including changes in reaction time, reaction temperature, solvent composition, and DAPMI and NaHSO_3_ concentrations. The initial derivatization time and temperature optimization revealed that a duration of 45 min and an 80 °C temperature provided the optimal labeling conditions to achieve the highest conversion of Neu5Ac to product **10** in aqueous solvent (Figure 2A,B). A DAPMI concentration of 20 mg/mL showed maximal labeling efficiency, with higher concentrations, e.g., 50 mg/mL, showing no significant improvements (Figure 2C). As such, a DAPMI concentration of 20 mg/mL (final concentration of 10 mg/mL) was selected for subsequent analysis. Regarding the NaHSO_3_ concentration, increasing levels up to 50–200 mM gave higher target signals; however, no significant difference was observed between these concentrations (Figure 2D). Thus, 200 mM NaHSO_3_ was deemed optimal for the following assays due to the reduced occurrence of byproducts. Screening the reaction using different labeling solvents revealed that water was the optimal solvent for the derivatization procedure (Figure 2E), with other solvents such as methanol ethanol, acetone, and THF suppressing the formation of DAPMI-Neu5Ac **10** to a large extent. Since many labeling tags are sensitive to light exposure and usually require storage conditions of −20 °C in the dark, we decided to evaluate the postderivatization stability of DAPMI-Neu5Ac **10** under different temperature and light conditions. The analysis showed that compound **10** exhibited negligible degradation over a storage period of 7 days, both in ambient or dark environments, and up to room-temperature conditions (Figure 2F) and minor degradation was observed under the optimized labeling conditions (see Supporting Information Section 2.1).

**Figure 2:**
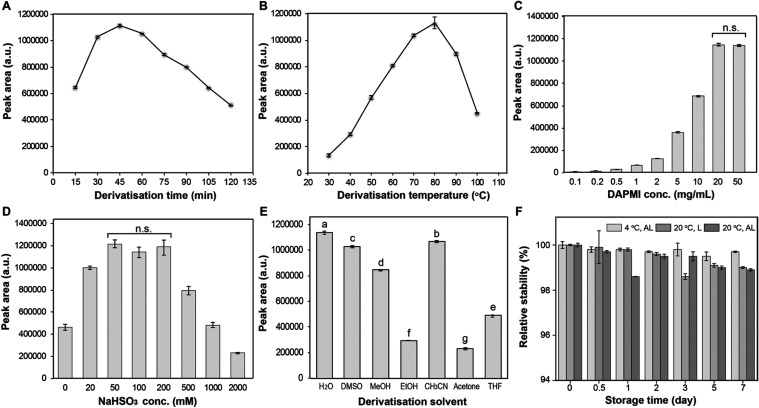
Optimization of DAPMI labeling efficiency. (A) Derivatization time optimization, (B) derivatization temperature optimization, (C) DAPMI concentration optimization, (D) NaHSO_3_ concentration optimization, (E) derivatization solvent screening, and (F) storage stability of compound **10** over a period of 7 days at different temperature and in the presence or absence of ambient light. Significance analysis of variance was performed by Duncan’s multiple range test (*p* < 0.05). Note: n.s. means no significance, different letters denote significant differences, AL: avoid light, L: light.

### Quantitative Analysis of Derivatized Sias

To compare the ionization efficiency of DAPMI **4** with the traditional Sia label OPD **1**, the limit of detection (LOD) and limit of quantification (LOQ) using Neu5Ac as standard Sia analyte were measured for both labeling reagents. For this purpose, Neu5Ac was derivatized with DAPMI **4** and OPD **1** at a preparative scale to give labeled derivatives **10** (8.8 mg) and **11** (2.2 mg), respectively, via an imine condensation reaction between the aryl amines with open form of Sias, specifically reacting with the acid and aldehyde functional groups of Sias (Scheme 1B and Supporting Information, Section 1). Next, serial dilutions (see Supporting Information Table S5) of the two analogues **10** and **11** were analyzed with UPLC-ESI-ToF-MS using a fluorescence detector (FLD). It is worth noting that Sias derivatization with DAPMI results in the formation of 2 condensates. The adducts revealed a 2:1 ratio being the trans the major isomer which displays a reduced steric hindrance between the nonreducing end of the sugar and the imidazolium moieties compared to the cis adduct. The isomers proved to be nonseparable using both silica gel and C-18 chromatography in both analytical and preparative scales. This feature does not impact the determination and quantification of Sias since both isomers have the same mass and were integrated together for Sias quantification. The calibration curves (see Supporting Information Section 1.6) were constructed by correlating the signal values from mass spectrometry and fluorescence analysis (see Supporting Information Figure S3) of various diluted samples with their corresponding known concentrations. The LOD (S/N = 3) of DAPMI-derivatized Neu5Ac **10** exhibited a remarkable 130-fold increase in ESI-MS detection sensitivity compared to OPD-derivatized Neu5Ac **11**. Similarly, the LOQ (S/N = 10) of **10** showed a 31-fold increase in ionization efficiency compared to OPD-derivatized Neu5Ac **11**. Furthermore, DAPMI-derivatized Neu5Ac **10** exhibited marginally higher fluorescence detection sensitivity in FLD analysis compared to OPD-derivatized Neu5Ac **11** when measured at their respective excitation and emission maxima (see SI Section 1 and Figure S1). Collectively, this result demonstrates the significant superiority of DAPMI **4** over standard OPD **1** for Sias detection (Table 1).

**Table 1:** Limit of Detection and Quantification of DAPMI-Neu5Ac **10** and OPD-Neu5Ac **11**

	ESI-MS^a^		fluorescence^a^	
compound	LOD (fmol)	LOQ (fmol)	LOD (fmol)	LOQ (fmol)
DAPMI-Neu5Ac (**10**)	2.55	28.78	380	1505
OPD-Neu5Ac (**11**)	326.15	890.67	512	1512

a Fluorescence intensities were measured for compound **10** at *E*_x_/*E*_m_ wavelength of 356/412 nm and for compound **11** at 354/416 nm.

### Sia Analysis from Biological Tissues

To further validate the applicability of DAPMI **4** for the efficient derivatization and qualitative and quantitative analysis of Sias in complex biological matrices, human serum, wild-type and CMAH knockout mouse liver, as well as mouse milk and wild-type mouse serum were selected as the target samples for analysis. The biological samples, including mouse livers, milk, serum, and human serum, were homogenized and treated with acetic acid to release the Sias. After centrifugation, the resulting supernatant was spin-dried and resuspended in ddH_2_O, which was then subjected to purification using an ion exchange column chromatography, followed by derivatization with DAPMI **4** to evaluate Sias composition and their relative content (see Supporting Information, Section 1, and Scheme S1). The quantitative analysis of Sias content in biological samples was performed with an external standard method with calibration curves. To further confirm the accuracy of Sias quantification, the spiking method with a known amount of DAPMI-Neu5Ac as the standard was also applied (see Supporting Information, Section 3), corroborating the accuracy of Sias quantification. The results revealed that the total Sias content in mouse milk, including samples from wild-type (WT, CMAH+/+, 38.89 ± 1.44 nmol), heterogeneous-type (HE, CMAH+/-, 46.82 ± 0.50 nmol), and homozygous-type (HO, CMAH–/–, 54.36 ± 1.45 nmol) mice, was higher than that in mouse liver (3.28 ± 0.13 nmol in HE mouse liver samples, and 6.2 ± 0.48 nmol in HO mouse liver samples) and serum (13.77 ± 0.61 nmol) (Figure 3B). In the liver samples from WT and HE mice, the ratio of Neu5Gc/Neu5Ac was relatively higher than in those from HO mice. This difference was attributed to the higher enzymatic regulation of CAMH in WT and HE mice, which facilitates the conversion of Neu5Ac to Neu5Gc and consequently results in a higher expression level of Neu5Gc in liver tissues.^45^ Moreover, the abundance of Neu5Gc in the milk was significantly lower than that in the liver tissue, accounting for ∼2% of total Sias in both WT and HE mouse milk. This finding is consistent with a previous study reporting 1–4% of Neu5Gc in the total Sia content of mouse milk.^46^ Similarly, the Neu5Gc contents in the HO mouse liver and milk samples were substantially lower, comprising only around 0.3% of the total Sias (Figure 3A–C). Additionally, a significant difference in the Neu5Gc/Neu5Ac ratio was observed between human and WT mouse serum, with human serum containing very small quantities of Neu5Gc compared with WT mouse serum (Figure 3D). This could be explained by CMAH inactivation in HO mice with dietary intake of Neu5Gc-rich foodstuffs, which leads to the metabolic incorporation of Neu5Gc in cellular glycans, allowing only trace amounts of Neu5Gc to be detected.^42,47^ It can be concluded that evolutionary selection plays a critical role in determining the preference for Neu5Gc or Neu5Ac in mammals and its distribution in various tissues. Particularly, pathogenic and endogenous pressures ultimately influence the balance of these two Sias which can be efficiently detected with the DAPMI labeling technology.

**Figure 3:**
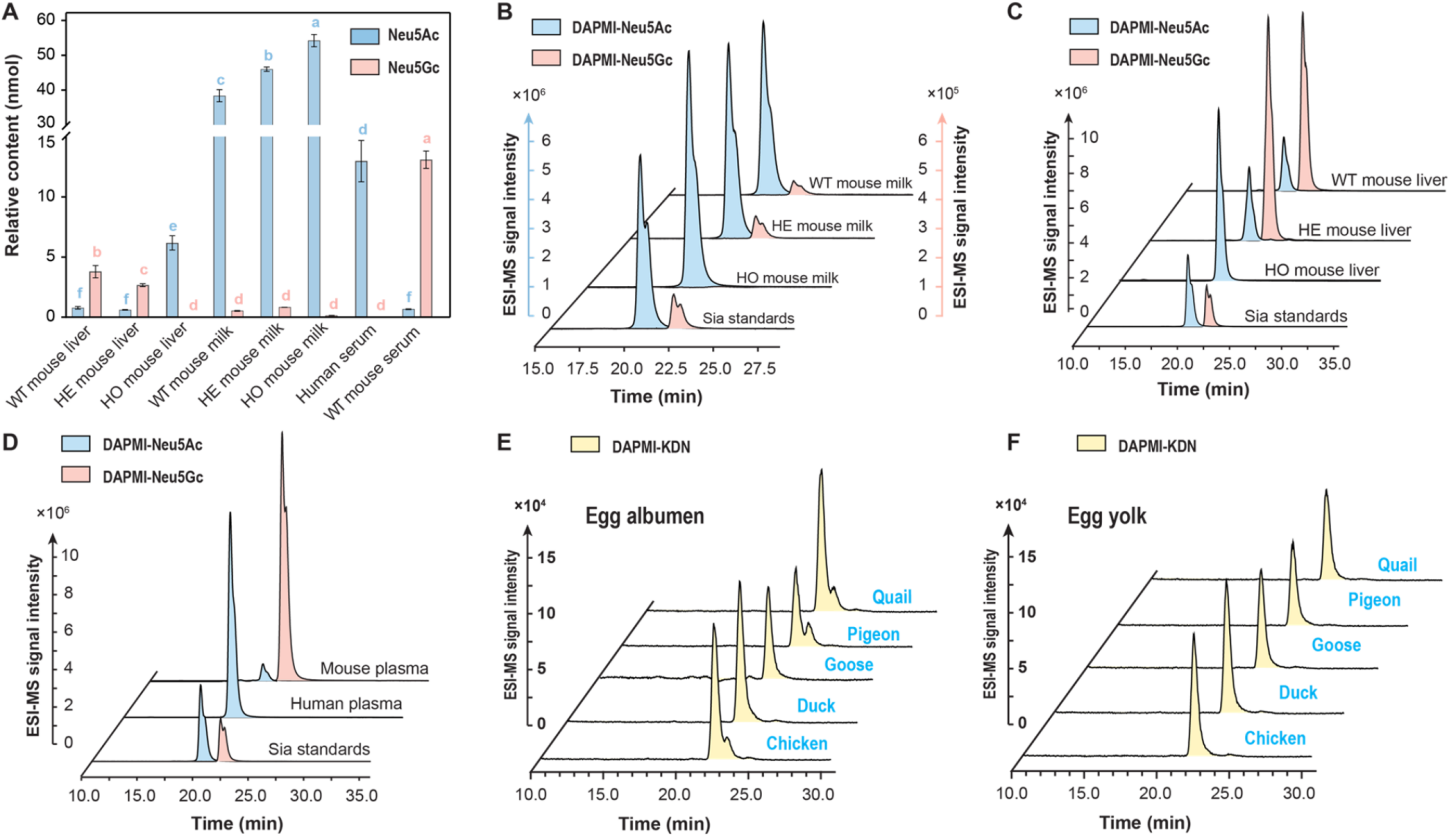
(A) Sia contents in different biological samples. All measurements were performed in triplicate. Different lowercase letters (blue color represents Neu5Ac, red color represents Neu5Gc) denote significant differences, which were assessed using Duncan’s multiple range test (*p* < 0.05). (B) ESI-MS profiles in single ion mode of DAMPI-derivatized Neu5Ac and Neu5Gc in mouse milk, (C) ESI-MS profiles in single ion mode of DAMPI-derivatized Neu5Ac and Neu5Gc in mouse liver, (D) ESI-MS profiles in single ion mode of DAMPI-derivatized Neu5Ac and Neu5Gc mouse and human serum, (E) ESI-MS profiles in single ion mode of DAMPI-derivatized KDN in egg albumen samples, and (F) ESI-MS profiles in single ion mode of DAMPI-derivatized KDN in egg yolk samples.

To improve the scope and confirm the feasibility of DAPMI **4** in various biological samples, five types of poultry egg samples, including egg yolks and egg albumens, were analyzed. The results showed that DAPMI is effective for determining the Sias content in poultry eggs. Notably, Neu5Ac and its derivative KDN were detected in the five egg samples (Figure 3E,F and Table S9). These findings confirmed that DAPMI has strong applicability for Sias detection across a wide range of biological samples.

## Conclusions

In summary, we have developed a novel bifunctional imidazolium Sia tag, DAMPI **4**, which features an *o*-phenylenediamine moiety for efficient derivatization of Sias, produces a fluorescent species upon Sia condensation. It also incorporates a cationic imidazolium group for excellent ionization efficiency in mass spectrometry analysis. The Sias derivatized with DAMPI exhibited comparable fluorescence intensity and significantly enhanced ionization efficiency in ESI-MS (a 130-fold improvement) when compared to commercial OPD labels, with a LOD as low as 2.55 fmol, demonstrating a significant improvement in detection sensitivity. Future studies are required to assess the potential of DAPMI Sias detection and quantification for the evaluation of health status of humans and possible correlations to severe diseases. However, in this proof-of-concept report, we have demonstrated the successful derivatization of Sias released from biological samples and confirmed the versatility of the novel ITag DAMPI for the efficient and sensitive analysis of Sias in complex matrices, enabling dual-mode MS and fluorescent detection as well as quantification in trace Sias samples.

## Supplementary Material



## Notes

Synthetic procedures, chromatographic methods, labeling procedures, chemical characterization, fluorometric spectra, and ^1^H and ^13^C NMR spectra for novel compounds (PDF)
