# Supplementary material for: Improved ESI-MS Sensitivity via an Imidazolium Tag (DAPMI-ITag) for Precise Sialic Acid Detection in Human Serum and CMAH-Null Mouse Tissues
Source: Anal Chem. 2025 Jun 9;97(24):12587–94. doi: 10.1021/acs.analchem.5c00752 (PMC12199228; doi:10.1021/acs.analchem.5c00752)
Supplement: Supplementary file 1 [file ac5c00752_si_001.pdf]

Supporting Information

Improved ESI-MS Sensitivity via an Imidazolium Tag (DAPMI-ITag) for Precise Sialic Acid Detection in Human Serum and CMAH-Null Mouse Tissues

Yao-Yao Zhang,[a,b,c+] Zi-Xuan Hu,[a+] Si-Yu Zhang,[a] Li Liu,[a] M. Carmen Galan,[c,*] Josef Voglmeir,[a,*] and Mattia Ghirardello[c,d,*]

[a] Glycomics and Glycan Bioengineering Research Center (GGBRC), College of Food Science and Technology, Nanjing Agricultural University, 1 Weigang, 210095 Nanjing (China)

[b] Lipid Technology and Engineering, School of Food Science and Engineering, Henan University of Technology, Lianhua Road 100, 450001 Zhengzhou (China)

[c] School of Chemistry, University of Bristol, Cantock's Close, BS8 1TS Bristol (UK)

[d] Institute of Biocomputation and Physics of Complex Systems (BIFI), University of Zaragoza, Calle Mariano Esquillor, Edificio I+D, 50018 Zaragoza (Spain)

[+] These authors contributed equally to this work.

*Correspondence should be addressed to josef.voglmeir@njau.edu.cn or m.c.galan@bristol.ac.uk or mghirardello@unizar.es

Table of contents

1. Materials and methods S2
    1.1 Materials S2
    1.2 Free Sia preparation and purification S2
    1.3 Synthesis of DAPMI tag S3
    1.4 Preparative-scale synthesis, purification and characterisation of DAPMI and OPD derivatized Neu5Ac S4
    1.5 Fluorometric profiling of DAPMI-Neu5Ac and OPD-Neu5Ac S9
    1.6 UPLC-ESI-MS analysis and quantification of DAPMI-Neu5Ac and OPD-Neu5Ac S10
2. Optimization of derivatization conditions S13
    2.1 Stability assay S13
3. Application of DAPMI tag in determination of Sias from biological samples S15
4. NMR Spectra S19
    4.1 NMR result of compound 6 .... S19
    4.2 NMR result of compound 7 .... S20
    4.3 NMR result of compound 9 .... S21
    4.4 NMR result of compound 4 .... S22
    4.5 NMR result of compound 10 .... S23

1. Materials and methods

1.1 Materials

3-Bromopropylamine hydrobromide, o-Phenylenediamine (OPD), 3,4-diaminobenzoic acid, *di-tert*-butyl decarbonate, *N*-methylimidazolium, potassium tetrafluoroborate (KBF_4), *N*-hydroxy succinimide (NHS) and *N*, *N*-diisopropylethylamine (DIPEA) were purchased from J&G chemicals (Nanjing, China); Dichloromethane (CH_2Cl_2), ethyl acetate (EtOAc), methanol (MeOH), acetonitrile (ACN), were obtained from General-Reagent Co. (Shanghai, China); Acetonitrile (ACN) used for HPLC analysis was purchased from Merck. (Nanjing, China). Wide-type mice were provided by Comparative Medicine Centre of Yangzhou University (China). CMAH knock-out mice were obtained commercially from Shanghai Model Organisms Center, Inc. (Shanghai, China), which were generated by removing 92 base pairs from exon 6 of the CMAH gene using a CRISPR/Cas9 strategy (10.1128/MCB.00379-07, 10.1016/j.cell.2014.09.014). Liver and milk samples were collected from both wild-type and CMAH knock-out mice. Human serum was supported under sterilized conditions from Prof. Josef Voglmeir. Mouse serum (wild-type) was obtained from Yuanye Bio-Technology Co., Ltd. (Shanghai, China); Other chemicals were obtained from commercial suppliers without further treatment. Procedures involving animal subjects have been approved by the Ethical Committee of the Experimental Animal Center of Nanjing Agricultural University in accordance to the National Guidelines for Experimental Animal Welfare (Ministry of Science and Technology, PR of China, 2006) with the animals housed in a SPF facility (Permission ID: SYXK-J-2011-0037).

1.2 Free Sia preparation and purification

The free Sia preparation from mouse liver, milk and serum was conducted following previously established procedures with minor modifications (10.3791/56030). The thawed mouse liver (50 mg) was homogenized in a 2 mL glassware grinder by adding 1.2 mL of 2 M acetic acid, after which it was transferred into a 2 mL Eppendorf tube. Gently thawed 50 μL of milk or serum was pipetted and followed by adding 1.2 mL of 2 M acetic acid as well. All samples were then hydrolyzed at 80 °C for 4 hours. After centrifugation at 4 °C, 12,000 g for 15 min, 1 mL of the supernatant was taken for centrifugal evaporation, after which it was resolubilized into a 700 μL aqueous solution, blended and ultrasonicated at room temperature for 2 hours. Subsequently, after centrifuging at 4 °C, 12,000 g for 15 min, 600 μL of the sample supernatant was transferred to the anion exchange resin (200 mg, Dowex1X8 100-200 Cl) which had been flowed through 2 mL of acetic acid (2 M) once and washed with 2 mL of ddH_2O three times, respectively. After adding 2 mL of ddH_2O, the resin was eluted by 1 mL of ammonium acetate (50 mM) and the dripped flow fraction was collected and spin-dried under vacuum (Scheme S1).

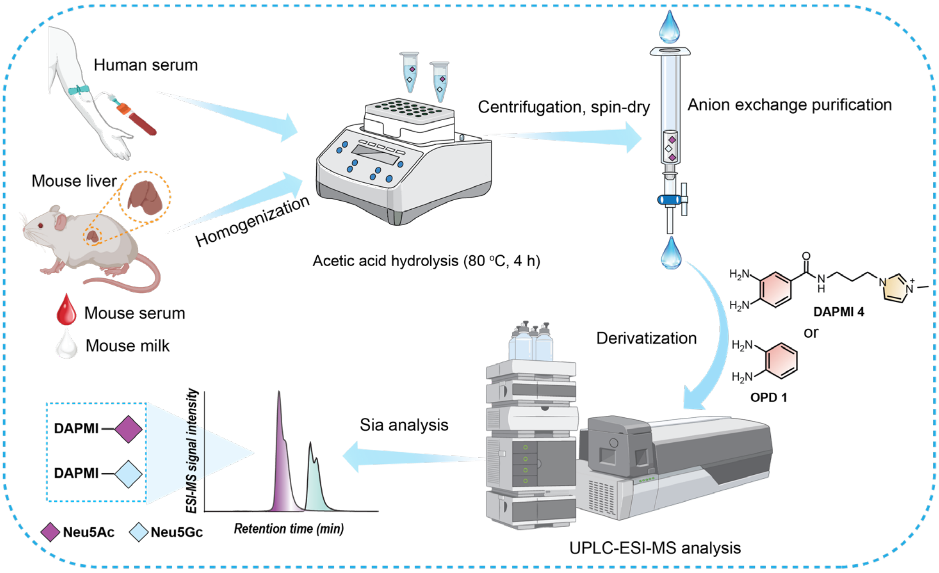

Scheme S1. Procedure of Sia preparation and analysis from biological samples.

1.3 Synthesis of DAPMI tag

The synthesis of DAMPI tag involves a sequential process as shown in Scheme S2.

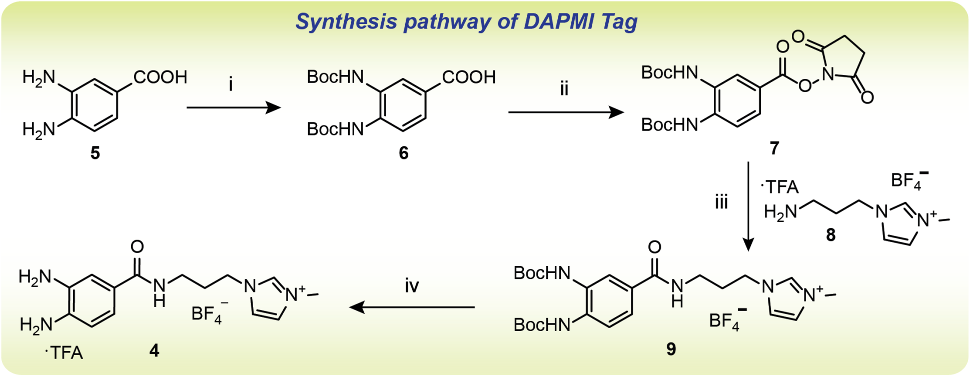

Scheme S2. Synthetic route for the synthesis of the Sia tag of DAMPI. Reagents and conditions. i) Boc_2O, TEA, H_2O/1,4-dioxane, 16 h, rt, 29%; ii) NHS, DCC, DMF, 16 h, rt, 92%; iii) **8**, DIPEA, DMF, 16 h, rt, 92%; iv) TFA, DCM, 2 h, rt, quant.

1.3.1 Synthesis of 3,4-bis((tert-butoxycarbonyl)amino)benzoic acid (6)

To a stirred suspension of 3,4-diaminobenzoic acid **5** (1.0 g, 6.57 mmol) in H_2O (20 mL), triethylamine (3.7 mL, 26.28 mmol) was added and the suspension turned immediately to a brown solution. A solution of di-*tert*-butyl decarbonate (3.2 g, 14.46 mmol) in 1,4-dioxane (20 mL) was added dropwise to the aqueous solution containing 3,4-diaminobenzoic acid at room temperature and the mixture was stirred for 16 h at room temperature. The solution was concentrated until about half of the initial volume under reduced pressure and poured into 75 mL of H_2O. The pH was adjusted to 4 using a saturated aqueous solution of citric acid causing the precipitation of the product. The mixture was extracted with EtOAc (3 x 100 mL) and the combined organic layers were then washed with H_2O (3 x 50 mL). The organic phase was dried with anhydrous $MgSO_4$, filtered and concentrated under reduced pressure. The residue was purified by column chromatography on silica gel (Hex/EtOAc 1:0 to 7:3, v/v containing a 0.5% v/v of AcOH) furnishing **6** (670 mg, 29% yield) as a white solid. ^{1}H NMR (400 MHz, Methanol-d_4) δ 8.09 (s, 1H, Ar), 7.81 (dd, J = 8.5, 2.0 Hz, 1H, Ar), 7.74 (d, J = 8.5 Hz, 1H, Ar), 1.55 (s, 9H, Boc), 1.54 (s, 9H, Boc). The NMR data are

in agreement to those reported in the literature (10.1080/15685551.2016.1257378).

1.3.2 Synthesis of 2,5-dioxopyrrolidin-1-yl 3,4-bis((tert-butoxycarbonyl)amino)benzoate (7)

To a stirred solution of **6** (310 mg, 0.88 mmol) in anhydrous DMF (4 mL), NHS (152 mg, 1.32 mmol) and DCC (271 mg, 1.32 mmol) were added as solids at room temperature and stirred for 16 h at room temperature. The suspension was filtered over a sintered funnel to remove the excess of insoluble dicyclohexylurea, washed with 5 mL of EtOAc and the filtrate was concentrated under reduced pressure. The residue was purified by column chromatography on silica gel (Hex/EtOAc 1:0 to 6:4, v/v) furnishing **7** (366 mg, 92% yield) as a white solid with minor traces of dicyclohexylurea. ^{1}H NMR (500 MHz, Methanol-d_4) δ 8.24 (s, 1H, Ar), 7.96 (d, J = 8.7 Hz, 1H, Ar), 7.90 (dd, J = 8.7, 2.1 Hz, 1H, Ar), 2.91 (s, 4H, NHS), 1.56 (s, 9H, Boc), 1.54 (s, 9H, Boc).
^{13}C NMR (126 MHz, MeOD) δ 170.5, 161.4, 154.4, 153.3, 129.0, 126.9, 126.5, 121.8, 119.8, 80.8, 80.5, 27.2, 27.1, 25.2. HRMS (ESI) m/z: Calcd for $C_{21}H_{28}N_3O_8$ $(M+H)^+$ 450.1871, found 450.1862.

1.3.3 Synthesis of 1-(3-aminopropyl)-3-methyl-1H-imidazol-3-ium tetrafluoroborate (8)

The synthesis of compound **8** refers to our previously established procedures (10.1016/j.carbpol.2024.122449).

1.3.4 Synthesis of 1-(3-(3,4-bis((tert-butoxycarbonyl)amino)benzamido)propyl)-3-methyl-1H-imidazol-3-ium tetrafluoroborate (9)

To a stirred solution of **7** (330 mg, 0.73 mmol) in anhydrous DMF (2 mL), compound **8** (124 mg, 0.365 mmol) and DIPEA (126 µL, 0.73 mmol) were added, and the reaction was stirred under inert atmosphere for 16 h at room temperature. The solution was concentrated under reduced pressure and the residue was purified by column chromatography on silica gel (MeOH/H_2O 1:0 to 95:5, v/v, containing 0.5% AcOH) to furnish **9** (198 mg, 92% yield) as a white wax with traces of AcOH. ^{1}H NMR (500 MHz, Methanol-d_4) δ 7.95 (d, J = 2.2 Hz, 1H, Ar), 7.74 – 7.68 (m, 2H, Ar, Im), 7.61 (dd, J = 8.5, 2.1 Hz, 1H, Ar), 7.57 (d, J = 2.0 Hz, 1H, Im), 4.31 (t, J = 6.8 Hz, 2H Im-CH_2), 3.91 (s, 3H, CH_3), 3.46 (t, J = 6.4 Hz, 2H, NHCH_2), 2.21 (p, J = 6.7 Hz, 2H, $CH_2Ci_2CH_2$), 1.55 (s, 18H, Boc).
^{13}C NMR (126 MHz, MeOD) δ 168.2, 154.6, 153.9, 136.7 (t, $J_{2H\text{-}13C}$ = 33.8 Hz), 134.5, 129.6, 129.4, 123.7, 123.6, 122.5, 122.2, 80.4, 80.3, 47.2, 36.1, 35.0, 29.7, 27.2, 27.2. HRMS (ESI) m/z: Calcd for $C_{24}H_{36}N_5O_5^+$ $(M)^+$ 474.2711, found 474.2698.

1.3.5 Synthesis of 1-(3-(3,4-diaminobenzamido)propyl)-3-methyl-1H-imidazol-3-ium tetrafluoroborate (4)

To a stirred suspension of **9** (81 mg, 0.14 mmol) in DCM (3 mL), TFA (3 mL) was added, and the solution was stirred for 2 h at room temperature. The mixture was concentrated under reduced pressure furnishing **4** (84 mg, quant. yield) as a brown solid used in the next step without further purification. ^{1}H NMR (500 MHz, Deuterium Oxide) δ 8.59 (s, 1H, Im), 7.48 (d, J = 2.0 Hz, 1H, Ar), 7.42 – 7.35 (m, 2H, Im, Ar), 7.27 (t, J = 1.9 Hz, 1H, Im), 6.91 (d, J = 8.5 Hz, 1H, Ar), 4.15 (t, J = 6.9 Hz, 2H, Im-CH_2), 3.68 (s, 3H, CH_3), 3.29 (t, J = 6.6 Hz, 2H, NHCH_2), 2.07 (p, J = 6.8 Hz, 2H, $CH_2CH_2CH_2$). ^{13}C NMR (126 MHz, D_2O) δ 168.9, 140.6, 135.0, 127.0, 124.7, 123.6, 122.7, 122.1, 119.4, 118.2, 47.2, 36.6, 35.5, 28.8. HRMS (ESI) m/z: Calcd for $C_{14}H_{20}N_5O^+$ $(M)^+$ 274.1662, found 274.1660.

1.4 Preparative-scale synthesis, purification and characterisation of DAPMI and OPD derivatized Neu5Ac

1.4.1 Synthesis and purification of DAPMI-derivatized Neu5Ac (10)

Preparative-scale synthesis of DAPMI-derivatized Neu5Ac (**10**) was performed in a 14 mL reaction system containing 40 mg DAMPI tag (**4**) (0.15 mmol), 31 mg Neu5Ac (0.1 mmol) and 83 mg $NaHSO_3$ (0.80 mmol) at 80

°C for 45 min, after which time TLC analysis (MeOH/H_2O/AcOH 8:2:0.5 v/v/v) revealed complete conversion of Neu5Ac. After freeze-drying, the product was rehydrated into a 1.2 mL aqueous solution. A 2 µL sample was injected into the Shimadzu HPLC-MS system equipped with UV (254 nm), fluorescence (Ex/Em = 356/412 nm), and ESI-MS detectors for the analysis of the product. The separation solvents consisted of ammonium formate (50 mM, pH 4.5, solvent A) and acetonitrile (solvent B), with solvent B increasing from 12% to 20% over 8 min, then to 95% in 1 min, and held for 2 min, at a total flow rate of 0.5 mL/min (Table S1). Purification of DAPMI-derivatized Neu5Ac was performed using an HPLC-SPD unit equipped with the preparative column (Cosmosil 5C18-MS-II, 20 mm ID × 250 mm), with a flow rate of 3 mL/min (Table S2). The collected DAMPI-Neu5Ac fraction was pooled, lyophilized furnishing **10** (8.8 mg, 16% yield) as a white solid as an inseparable 2:1 mixture of trans/cys isomers. ^{1}H NMR (500 MHz, Deuterium Oxide) δ 7.92 (d, *J* = 2.0 Hz, 1H, Ar), 7.77 – 7.66 (m, 1H, Ar), 7.56 – 7.51 (m, 1H, Ar), 7.47 – 7.43 (m, 1H, Im), 7.34 – 7.24 (m, 2H, Ar, Im), 4.32 – 4.15 (m, 4H, Im-CH_2, H-4^{SA}, H-5^{SA}), 3.78 – 3.65 (m, 6H, CH_3, H-6^{SA}, H-9a^{SA}, H-9b^{SA}), 3.49 – 3.37 (m, 3H, NHCH_2, H-7^{SA}), 3.33 – 3.27 (m, 1H, H-8^{SA}), 3.07 – 3.00 (m, 1H, H-3a^{SA}), 2.97 – 2.86 (m, 1H, H-3b^{SA}), 2.22 – 2.13 (m, 2H, $CH_2CH_2CH_2$), 2.00 (s, 3H, Ac). ^{13}C NMR (126 MHz, D_2O) δ 174.9, 174.9, 174.2, 169.0, 168.9, 168.9, 158.9, 158.2, 157.1, 135.9, 134.9, 134.5, 133.9, 131.4, 128.8, 128.5, 127.9, 126.6, 126.5, 123.6, 123.6, 122.2, 122.2, 117.2, 116.0, 80.2, 80.1, 74.6, 74.6, 73.2, 73.2, 66.9, 60.5, 52.3, 52.3, 47.3, 47.3, 47.2, 36.8, 36.8, 35.5, 34.8, 34.7, 28.6, 28.7, 21.9, 21.9. HRMS (ESI) m/z: Calcd for $C_{25}H_{35}N_6O_8^+$ (M)$^+$ 547.2405, found 547.2395.

Table S1 UPLC elution procedure of C18 reverse phase separation for DAPMI-derivatized Neu5Ac.

Time (min)	Module	Command	Value
0	Pumps	Solvent B Conc.	12
8	Pumps	Solvent B Conc.	20
9	Pumps	Solvent B Conc.	95
11	Pumps	Solvent B Conc.	95
12	Pumps	Solvent B Conc.	12
20	Pumps	Solvent B Conc.	12
20	Controller	stop	

Table S2 UPLC elution procedure of preparative C18 reverse phase separation for DAPMI-Neu5Ac.

Time (min)	Module	Command	Value
0	Pumps	Solvent B Conc.	8
45	Pumps	Solvent B Conc.	12
50	Pumps	Solvent B Conc.	95
60	Pumps	Solvent B Conc.	95
65	Pumps	Solvent B Conc.	8
80	Pumps	Solvent B Conc.	8
80	Controller	stop	

1.4.2 Synthesis and purification of OPD-derivatized Neu5Ac (11)

A total of 5.8 mL solution containing 50 mg Neu5Ac (0.16 mmol), 26 mg OPD **1** (0.24 mmol) and 54 mg $NaHSO_3$ (0.52 mmol) was reacted at 80 °C for 45 min, after which time TLC analysis (MeOH/H_2O/AcOH 8:2:0.5 v/v/v) revealed complete conversion of Neu5Ac. Benzaldehyde (2 eqv., v/v) was added and incubated at 37 °C for 30 min to remove excess OPD. Then, 5 mL of water was added and vortexed for 2 min, resulting in two phases after centrifugation at 4 °C, 12,000 rpm for 10 min. The top aqueous phase was harvested for spin dry under vacuum and re-dissolved in 1 mL of water for purification using solid phase extraction resin (Supelclean^{TM} ENVI^{TM}-18 SPE, 500 mg). The SPE column was washed with 3 mL of acetonitrile followed by 3 mL of ddH_2O, and then 1 mL of sample was loaded on the top of SPE column. The sample was eluted with 3 mL each of 0%, 10%, 50% and 100% (v/v) acetonitrile respectively. The eluted fraction was collected for TLC screening using n-butanol/AcOH/H_2O (5: 3: 2, v: v: v) as the separation solvents and DPA staining (5.91 mmol diphenylamine and 10.75 mmol aniline in 50 mL acetone and 5 mL phosphoric acid) for visualization. A 2 µL sample was subjected into an LCMS-8040 system (Shimadzu inc., Japan) coupled with a reversed-phase column (Cosmosil 5C18 MS-II 4.6×250 mm, Nacalai Inc., Japan). The analytes were identified using HPLC equipped with UV (254 nm), fluorescence (Ex/Em = 354/416 nm), and ESI-MS detectors with a mobile phase containing H_2O (solvent A), acetonitrile (solvent B), and methanol (solvent C) at a flow rate of 0.5 mL/min. The separation followed a linear gradient of 5-25% B and C for 15 min, 25-40% B and C for 5 min and held at 40% for 4 min (Table S3). Mass detection was set in positive ion mode with the m/z scan range of 100-700 Da and the target OPD-Neu5Ac fraction was spin-dried and weighed for further characterisation (Table S4).

Table S3 UPLC elution procedure of C18 reverse phase separation for OPD-Neu5Ac.

Time (min)	Module	Command	Value
0	Pumps	Solvent B Conc.	5
0	Pumps	Solvent C Conc.	5
15	Pumps	Solvent B Conc.	25
15	Pumps	Solvent C Conc.	25
20	Pumps	Solvent B Conc.	40
20	Pumps	Solvent C Conc.	40
24	Pumps	Solvent B Conc.	40
24	Pumps	Solvent C Conc.	40
28	Pumps	Solvent B Conc.	5
28	Pumps	Solvent C Conc.	5
32	Pumps	Solvent B Conc.	5
32	Pumps	Solvent C Conc.	5
32	Controller	stop	

Table S4 Parameters of mass spectrometric detection of Neu5Ac labelled by DAPMI and OPD.

ESI-MS condition	Parameter
Mass spectrometer	LCMS-8040
Ion source	ESI
Ion scanning mode	Positive ion scan mode
Interface temperature	350 °C
Desolvation tube temperature	250 °C
Heating module temperature	200 °C
Nebulizer gas flow rate	3 L/min
Drying gas flow rate	15 L/min
Detector voltage	1.5 kV

Neu5Ac labelled with DAPMI and OPD can be identified as $[M]^+$ ions (m/z=547.0), and $[M+Na]^+$ ions (m/z=404.0), respectively in ESI-MS (Figure S2).

1.5 Fluorometric profiling of DAPMI-Neu5Ac and OPD-Neu5Ac

The purified DAPMI-Neu5Ac and OPD-Neu5Ac were prepared as a solution in water (final concentration 1 mM), respectively. A total of 100 μL of each solution were transferred to a Costar black flat bottom 96-well plate for fluorescence scanning using a Spark Fluorescence Scanner (Thermofisher Inc., USA). For the emission spectrum, the excitation wavelength was set to 350 nm, and the emission wavelength was scanned from 400 nm to 500 nm. For excitation spectrum, the emission wavelength was maintained at 410 nm, and the emission wavelength was scanned from 300 nm to 375 nm. The excitation and emission spectra of these two compounds were recorded as shown in Figure S1.

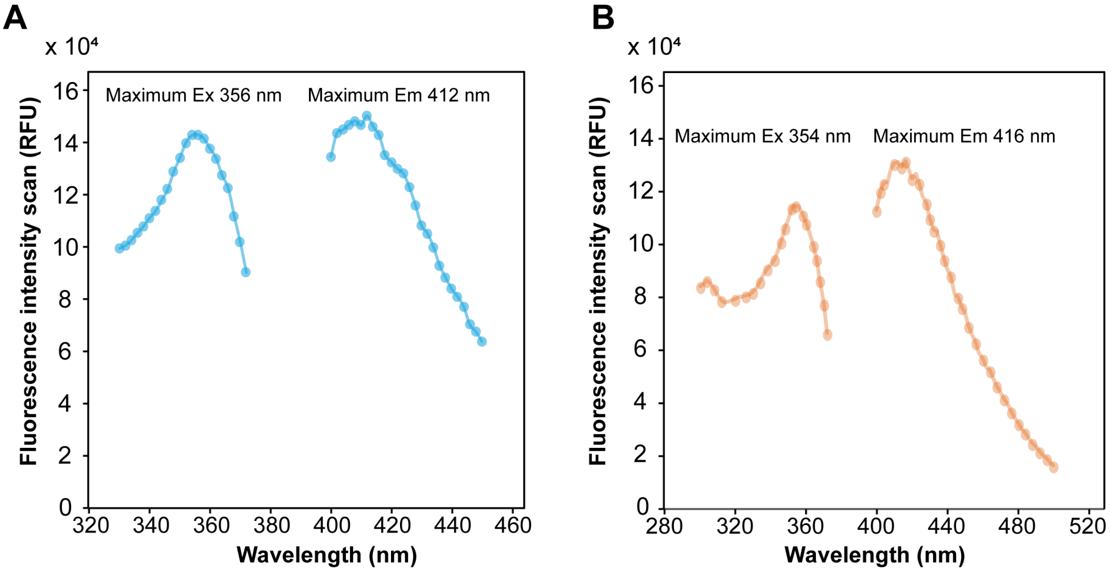

Figure S1. Emission and excitation spectra of the DAPMI-derivatized Neu5Ac (**A**), and OPD-derivatized Neu5Ac (**B**).

1.6 UPLC-ESI-MS analysis and quantification of DAPMI-Neu5Ac and OPD-Neu5Ac

To compare the fluorescence and ionization efficiency of Neu5Ac labelled with DAPMI **4** and commonly used OPD **1**, the absolute quantification of sample concentration based on the gravimetric determination of pure samples is essential. The serial dilutions of Neu5Ac labelled with DAPMI and OPD (ranging from 1 nM-50 μM) were then subject to UPLC-ESI-MS analysis and compared with the LOD and LOQ values. Shimadzu LCMS 8040 system (Shimadzu Corporation, Kyoto, Japan), consisting of an LC-30AD pump equipped with a low-pressure gradient mixing unit, a SIL-30AC autosampler, an RF-20Axs fluorescence detector, and an ESI mass spectrometric detector, was used for quantitative analysis (Table S5). 2 μL of the analytes, comprising two compounds, were separated on a reversed-phase HPLC column (Cosmosil 5C18 MS-II 4.6×250 mm, Nacalai Inc., Japan) at a constant flow rate of 0.5 mL/min with fluorometric detection (Table S1 and S3). The calibration curves (Figure S4) were established based on the correlation between the signal values in mass spectrometry and fluorescence analysis of different diluted samples (Figure S3) and their exact concentrations.

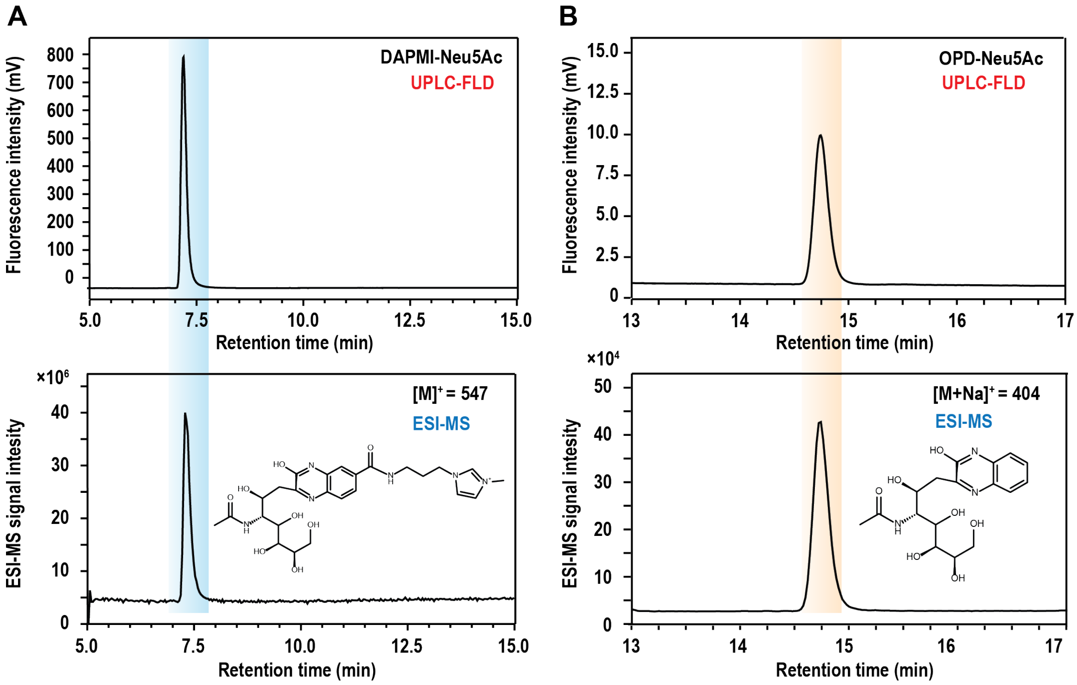

Figure S2. Identification of DAMPI-Neu5Ac (**A**) and OPD-Neu5Ac (**B**) by UPLC-FLD-MS analysis.

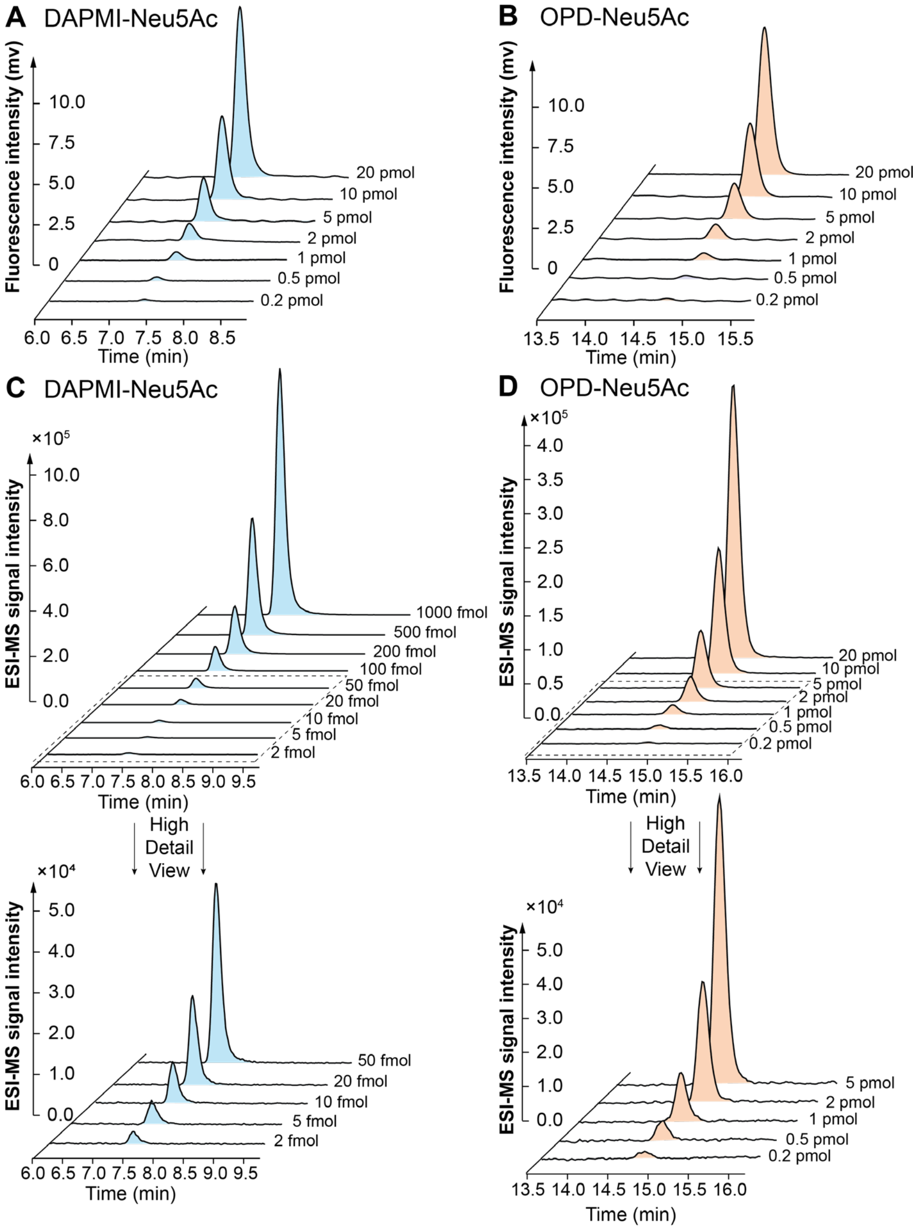

Figure S3. Comparison of fluorescence intensities and extracted ion count (EIC) chromatograms profiles of DAPMI and OPD labelled Neu5Ac. (**A**) Fluorescence intensity profile of different concentrations of DAPMI-Neu5Ac. (**B**) Fluorescence intensity profile of different concentrations of OPD-Neu5Ac. (**C**) EIC chromatogram of different concentrations of DAPMI-Neu5Ac. (**D**) EIC chromatogram of different concentrations of OPD-Neu5Ac.

Table S5 The linear calibration ranges and fitting coefficient of DAPMI-Neu5Ac and OPD-Neu5Ac.

Compounds	ESI-MS		Fluorescence	
	Linear range (fmol)	R^2	Linear range (pmol)	R^2
DAPMI-Neu5Ac	2-500	0.9993	0.2-20	0.9982
OPD-Neu5Ac	200-5000	0.9973	0.2-10	0.9966

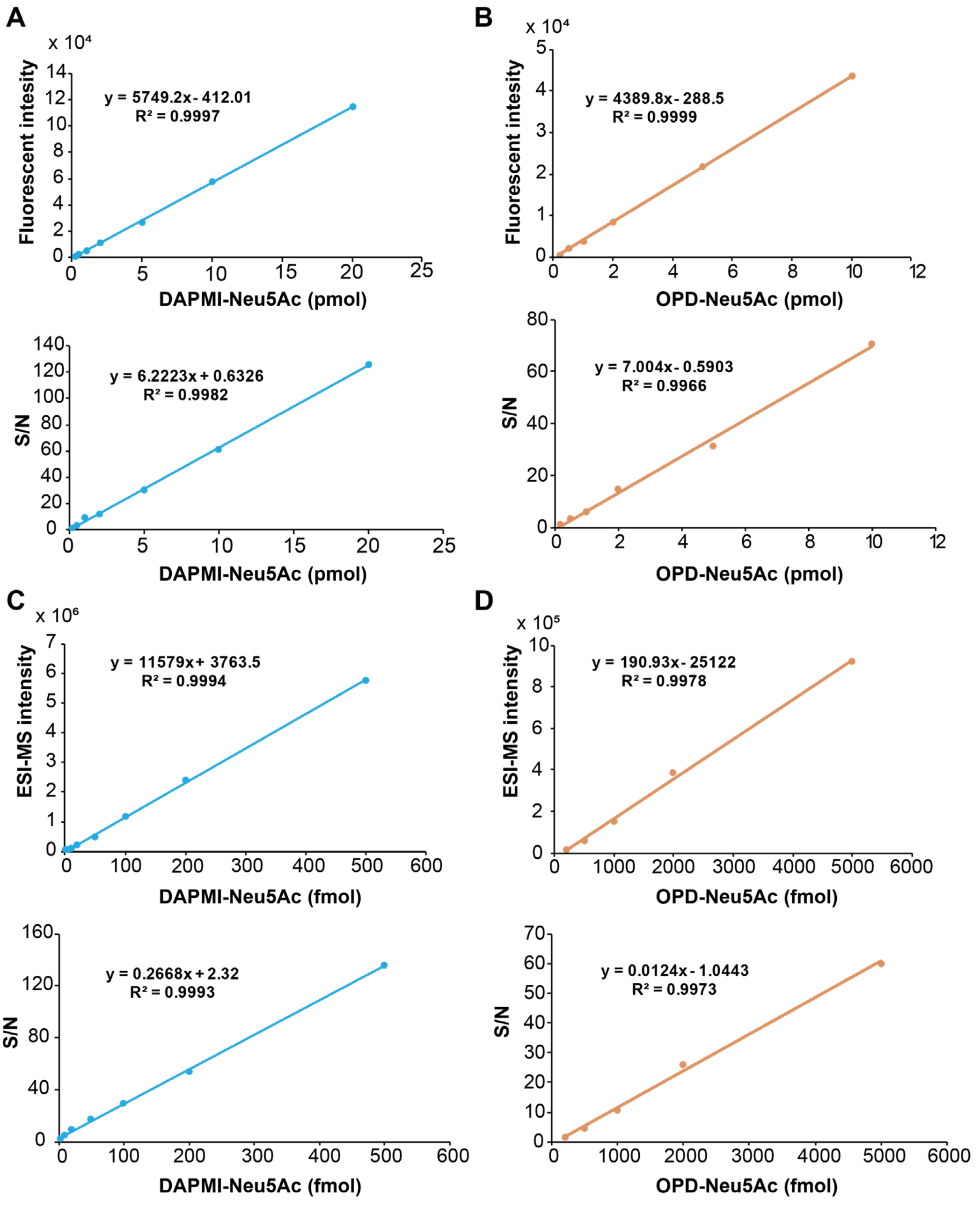

Figure S4. Calibration curves of fluorescence intensity for DAPMI-Neu5Ac (**A**), OPD-Neu5Ac (**B**); and calibration curves of ESI-MS intensity for DAPMI-Neu5Ac (**C**) and OPD-Neu5Ac (**D**).

2. Optimization of derivatization conditions

Optimization of derivatization conditions, including derivatization time, derivatization temperature, DAPMI concentration, sodium bisulfite ($NaHSO_3$) concentration and derivatization solvent was performed based on a 20 µL reaction volume composed of 2 µL of Neu5Ac (50 mM), 8 µL sodium bisulfite (500 mM) and 10 µL of DAPMI (20 mg/mL) aqueous solutions, which was derivatized at 80 °C for 45 min. For derivatization time and temperature optimization, the reaction was sampled at different times (15 min, 30 min, 45 min, 60 min, 75 min, 90 min, 105 min and 120 min) and temperatures (30 °C, 40 °C, 50 °C, 60 °C, 70 °C, 80 °C, 90 °C and 100 °C). For DAPMI and sodium bisulfite concentration optimization, the reaction was derivatized with different DAPMI concentration (0.1 mg/mL, 0.2 mg/mL, 0.5 mg/mL, 1 mg/mL, 2 mg/mL, 5 mg/mL, 10 mg/mL, 20 mg/mL, and 50 mg/mL) and $NaHSO_3$ concentration (0 mM, 20 mM, 50 mM, 100 mM, 200 mM, 500 mM, 1000 mM, and 2000 mM). For the optimization of the derivatization solvent, 20 µL reaction volume were treated with DAPMI (final concentration of 10 mg/mL) dissolved in different solvents (H_2O, DMSO, MeOH, EtOH, CH_3CN, Acetone and THF). All samples were diluted 50 times before HPLC analysis.

To evaluate the storage stability after DAPMI derivatization, 20 µL of the labelling reaction were diluted 50-fold, and a sample of 100 µL of the diluted solution was stored either at 4 °C in the absence of light, or at 20 °C in presence of environmental light, or at 20 °C in absence of environmental light. HPLC analysis was carried out using C18 reverse phase column (Cosmosil 5C18 MS-II 4.6×250 mm, Nacalai Inc., Japan) and fluorescent detector (Ex/Em=356 nm/ 412 nm). The elution phases consisted of solvent A (50 mM ammonium formate, pH 4.5) and solvent B (acetonitrile) followed the elution procedure in Table S1.

2.1 Stability assay

To analyze the stability of derivatization reagent and the biological samples, the derivatization reagent (DAPMI), Neu5Ac, and DAPMI-Neu5Ac were incubated respectively under the optimal derivatization conditions (Reaction temperature: 80 °C with 0.2 M $NaHSO_3$). The samples were taken at different incubation times (0, 5, 15, 30 and 45 min) and analyzed using HPLC-ESI-MS. The results showed that the derivatization reagent (DAPMI), Neu5Ac, and DAPMI-Neu5Ac were relatively stable under the optimal labelling conditions (Figure S5), further confirming the robust of DAPMI in the Sias derivatization and analysis in biological samples.

The stability test was performed by UPLC coupled with a fluorescent detector (Ex/Em=356 nm/ 412 nm, for DAPMI-Neu5Ac) and a UV detector (254 nm, for DAPMI), and 5 µL of each sample was injected and separated with reverse-phase C18 chromatography column (Cosmosil 5C18 MS-II 4.6×250 mm, Nacalai Inc., Japan) at a constant flow rate of 0.5 mL/min. The elution buffers were ammonium formate (50 mM, pH 4.5, solvent A) and acetonitrile (solvent B), following the elution procedure described in Table S1. As for Neu5Ac stability test, a 10 µL sample was separated with reverse-phase C18 column and recorded with a Shimadzu 8040 ESI-MS detector in positive single ion mode ($[M+H]^+$=310). The elution condition was modified on the basis of DAPMI-Neu5Ac separation procedure (see Table S6) with solvent B increasing from 12% to 20% over 5 min, then to 95% in 1 min, and held for 1 min, at a total flow rate of 0.5 mL/min. HPLC or ESI-MS spectra integration of the product peak was used as the key monitoring parameter for stability evaluation. Within, the stability of 0 min sample point (P_0) was set as 100%, and the relative stability of other samples (P) was calculated by the following formula: $\text{Relative stability} = \frac{P}{P_0} * 100\%$.

Table S6 UPLC elution procedure of C18 reverse phase separation for Neu5Ac.

Time (min)	Module	Command	Value
0	Pumps	Solvent B Conc.	12
5	Pumps	Solvent B Conc.	20
6	Pumps	Solvent B Conc.	95
7	Pumps	Solvent B Conc.	95
8	Pumps	Solvent B Conc.	12
10	Pumps	Solvent B Conc.	12
10	Controller	stop	

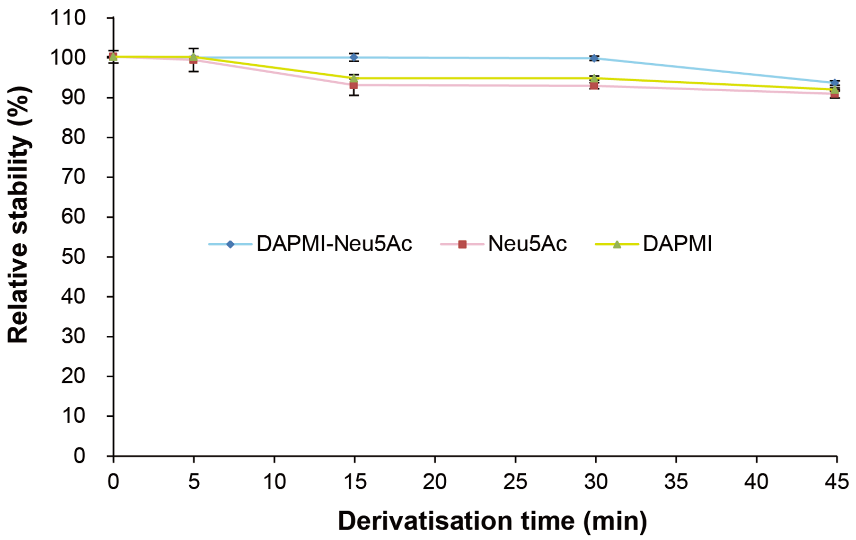

Figure S5. Stability test of DAPMI-Neu5Ac, Neu5Ac, and DAMPI under the optimal labelling conditions.

3. Application of DAPMI tag in determination of Sias from biological samples

A 20 μL of DAPMI tag solution (10 mg/mL in 0.2 M sodium bisulfite) was added to the dried Sias residue derived from biological samples (human serum, mouse liver, mouse milk, and mouse serum) for derivatisation at 80 °C for 45 min, followed by centrifugation treatment (4 °C, 12,000 rpm,15 min). 5 μL of the tagged solution were injected into a Acquity BEH Amide Column (2.1×150 mm, 1.7 μm, Waters, Ireland) at 60 °C and analyzed with a Shimadzu UPLC-FLD-MS system. The elution solvents comprised solvent A (50 mM ammonium formate, pH 4.5) and solvent B (acetonitrile), with a total flow rate is 0.4 mL/min. The separation gradient was performed as follows: 88% B from 0-1.5 min, 88% to 70% B from 1.5-35 min (Table S7). The spectra were recorded using an RF-20Axs fluorescence detector (λ_{ex} = 356 nm, λ_{em} = 412 nm) and an 8040 ESI-ToF detector (positive single ion mode).

The quantitative analysis of Sias content in biological samples was performed with external standard method with calibration curves. To further confirm the accuracy, the spiking method with known amount of DAPMI-Neu5Ac as the standard was also applied. In the spiking method, the biological samples (WT mouse milk, HE mouse milk, and WT mouse serum) were prepared and derivatized at 80 °C for 45 min, followed by centrifugation treatment (4 °C, 12,000 rpm,15 min). Then, the pure compound of DAMPI-Neu5Ac with known concentration (200 nM) was used as the standard substance (spike) and added in the derivatized samples with varying volumes, resulting in spiked samples with different analyte concentrations. The samples before spiking and after spiking were analyzed using HPLC-ESI-MS. The calibration curves were established according to the concentration of the standard solution and corresponding response signals. The concentration of Sias in the biological samples were calculated using the constructed standard curves. It can be seen from the results, there is no significant difference between the external standard method and the spiking methods, further validating the accuracy of the detection method in this study (Figure S6 and Table S8).

To improve the scope and confirm the feasibility of DAPMI **4** in various biological samples, five types of poultry egg samples, including egg yolks and egg albumens, were analyzed. A 20 μL of DAPMI tag solution (10 mg/mL in 0.2 M sodium bisulfite) was added to the dried Sias residue derived from poultry egg samples (chicken yolk and albumen, duck yolk and albumen, quail yolk and albumen, goose yolk and albumen, and pigeon yolk and albumen) for derivatisation at 80 °C for 45 min, followed by centrifugation treatment (4 °C, 12,000 rpm,15 min). 5 μL of the tagged solution were injected into a Acquity BEH Amide Column (2.1×150 mm, 1.7 μm, Waters, Ireland) at 60 °C and analyzed with a Shimadzu UPLC-FLD-MS system. The Neu5Ac and KDN content in different egg samples were determined using spiking method, which can be seen in Table S9.

To further confirm the applicability of DAPMI in derivatization of Sias, various types of Sias, including Neu5Ac, Neu5Gc, Neu5Fo, KDN, 5-*epi*-KDN, and 5, 7-di-*epi*-KDN were used as the standard compounds. The standard compounds (25 nmol) were derivatized with 20 μL of DAPMI tag solution (10 mg/mL in 0.2 M sodium bisulfite) at a reaction temperature of 80 °C for 45 min, followed by centrifugation treatment (4 °C, 12,000 rpm,15 min). The standard samples were diluted 50-fold and then analyzed using HPLC-ESI-MS. It can be seen from the results, DAPMI demonstrated its high applicability for various type of Sias derivatization and detection (Figure S7 and Table S10).

Table S7 UPLC elution procedure of HILIC separation for DAPMI derivatized samples.

Time (min)	Module	Command	Value
0	Pumps	Solvent B Conc.	88
1.5	Pumps	Solvent B Conc.	88
35	Pumps	Solvent B Conc.	70
35.5	Pumps	Solvent B Conc.	0
36	Pumps	Solvent B Conc.	0
36.5	Pumps	Solvent B Conc.	88
45	Pumps	Solvent B Conc.	88
45	Controller	stop	

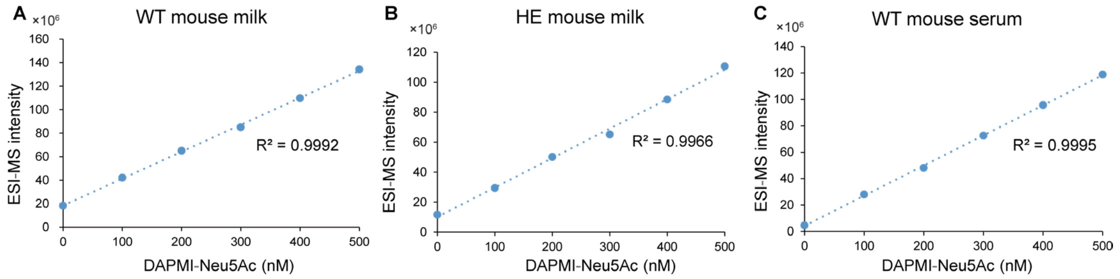

Figure S6. The calibration curves for the quantitative analysis of Sia in WT mouse milk (**A**), HE mouse milk (**B**), and WT mouse serum (**C**) using spiking method.

Table S8 Sias content in biological samples using external standard method and the spiking methods.

Samples	Neu5Ac content (nmol)		Neu5Gc content (nmol)	
	Spiking method	External standard method	Spiking method	External standard method
WT mouse milk	40.10 [a]	38.36 [a]	0.39 [d]	0.53 [d]
HE mouse milk	51.71 [b]	45.99 [b]	0.76 [e]	0.83 [e]
WT mouse serum	0.77 [c]	0.67 [c]	11.55 [f]	13.10 [f]

Note: The difference between two quantitative analysis methods was evaluated using a paired samples t-test. The same lowercase letters indicate that there is no significant difference between the two testing methods ($p < 0.05$).

Table S9 The Neu5Ac and KDN content in different egg samples.

Samples	DAPMI-Neu5Ac (nmol)	DAPMI-KDN (nmol)
Chicken yolk	399.40	5.85
Chicken albumen	440.33	4.93
Duck yolk	341.07	4.91
Duck albumen	118.49	5.24
Quail yolk	376.76	5.07
Quail albumen	330.45	5.66
Goose yolk	425.86	5.89
Goose albumen	97.83	7.81
Pigeon yolk	506.21	5.10
Pigeon albumen	2499.10	5.92

Table S10 The structure and molecular weight of various Sias.

Sias	Structure	Molecular weight after DAPMI derivatisation (Da)
Neu5Ac	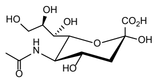	547
Neu5Gc	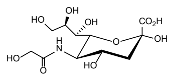	563
Neu5Fo	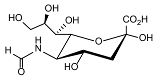	533
KDN	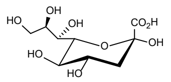	506
5-*epi*-KDN	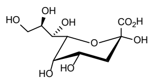	506
5,7-di-*epi*-KDN	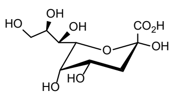	506

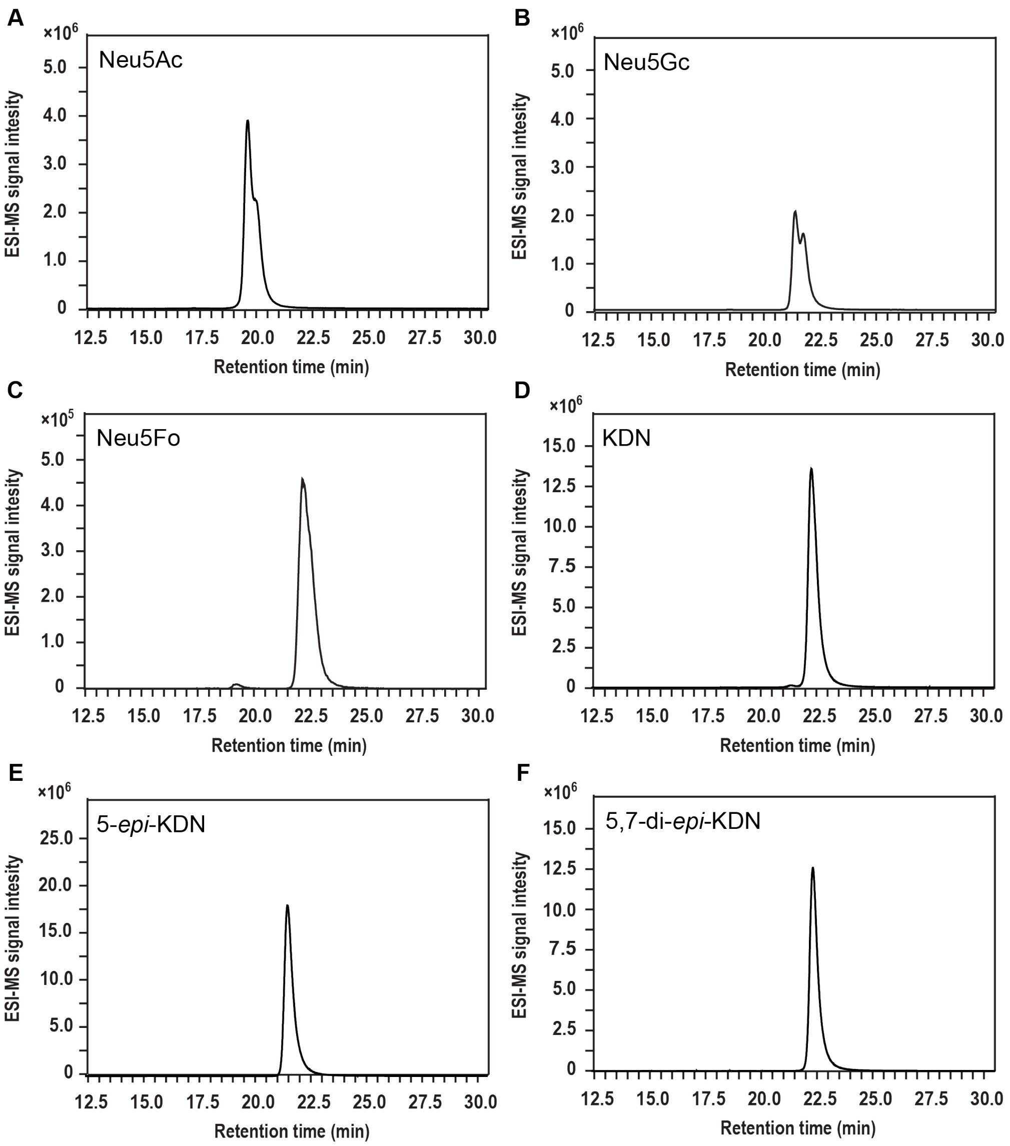

Figure S7. ESI-MS profiles in single ion mode of DAPMI-Neu5Ac (**A**), DAPMI-Neu5Gc (**B**), DAPMI-Neu5Fo (**C**), DAPMI-KDN (**D**), DAPMI-5-*epi*-KDN (**E**), and DAPMI-5,7-di-*epi*-KDN (**F**).

4. NMR Spectra

4.1 NMR result of compound 6

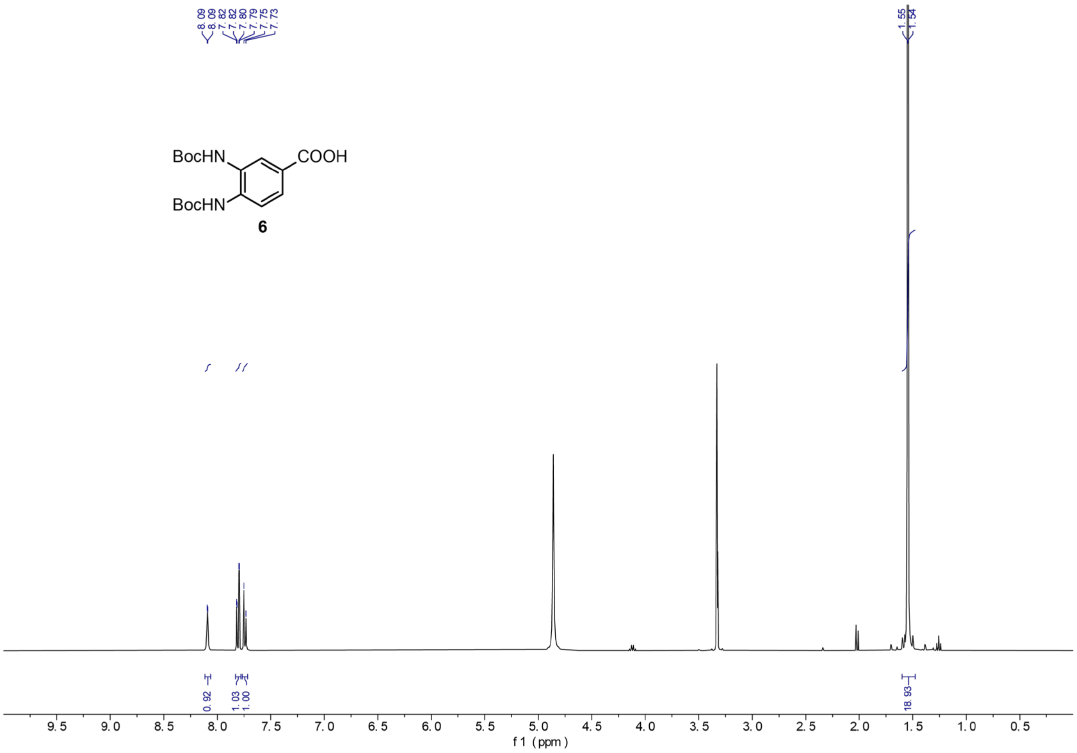

Figure S8. ^{1}H NMR result of compound **6**.

4.2 NMR result of compound 7

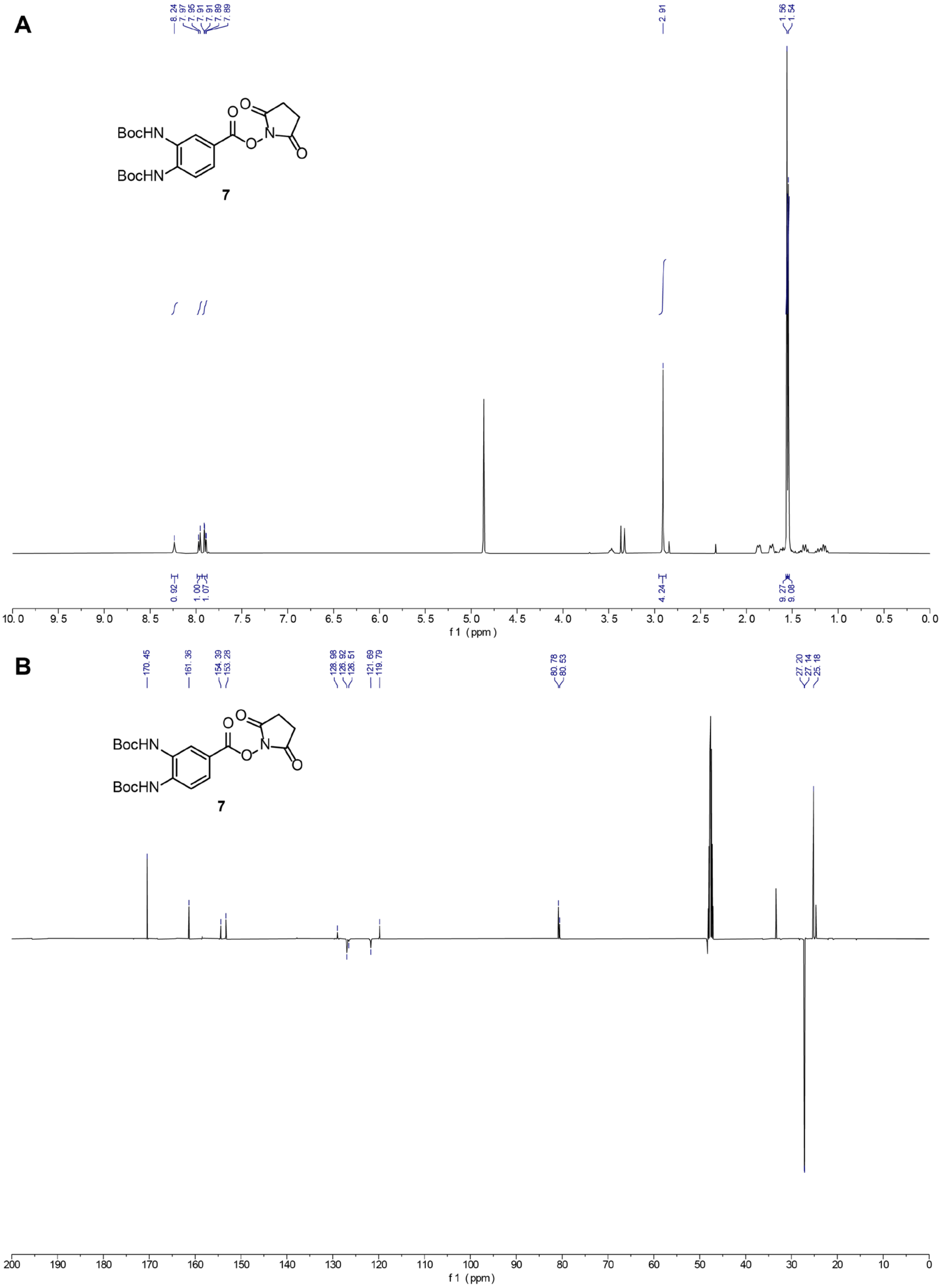

Figure S9. ^{1}H NMR (**A**) and ^{13}C APT NMR (**B**) results of compound **7**.

4.3 NMR result of compound 9

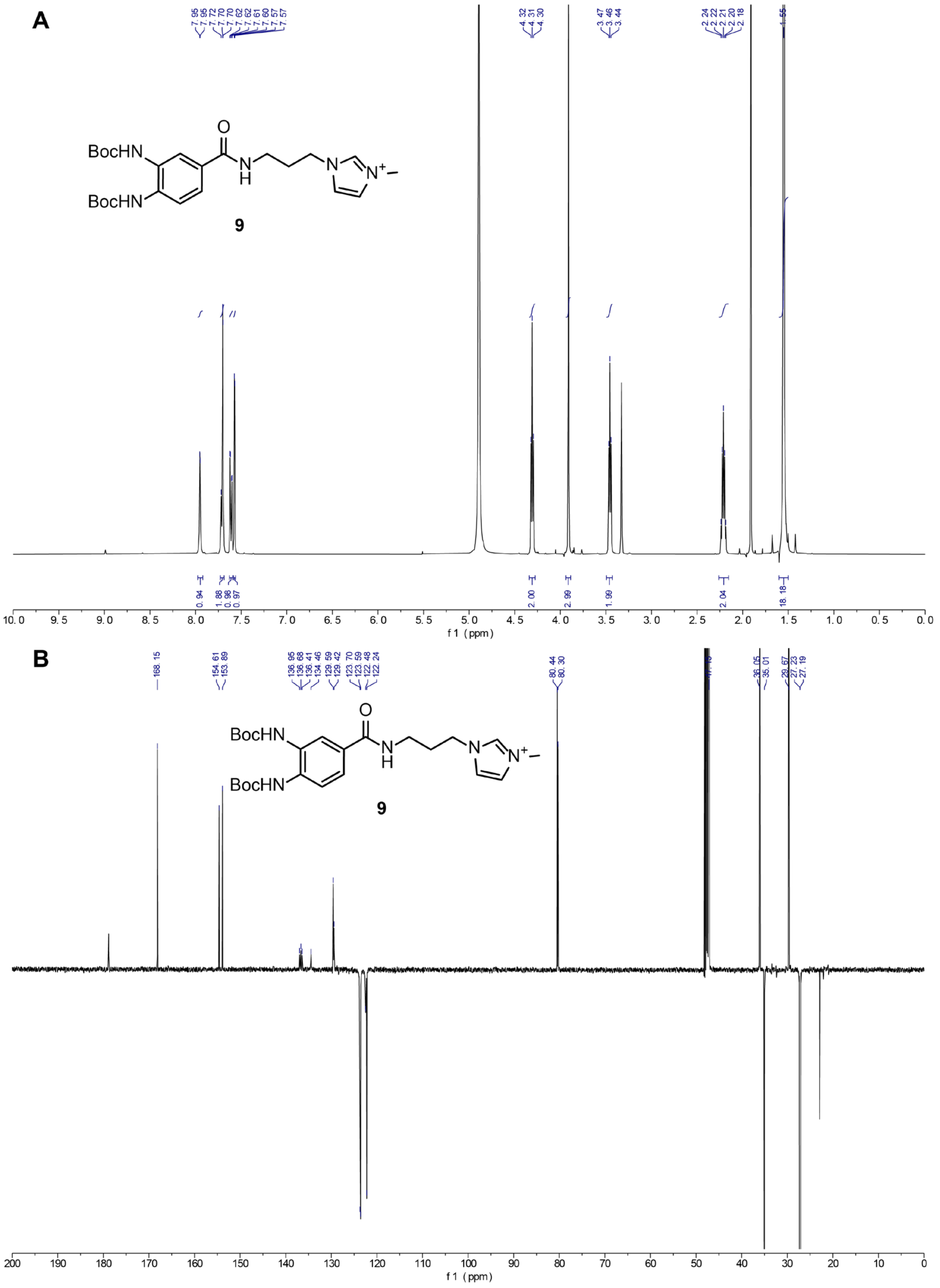

Figure S10. ^{1}H NMR (**A**) and ^{13}C APT NMR (**B**) results of compound **9**.

4.4 NMR result of compound 4

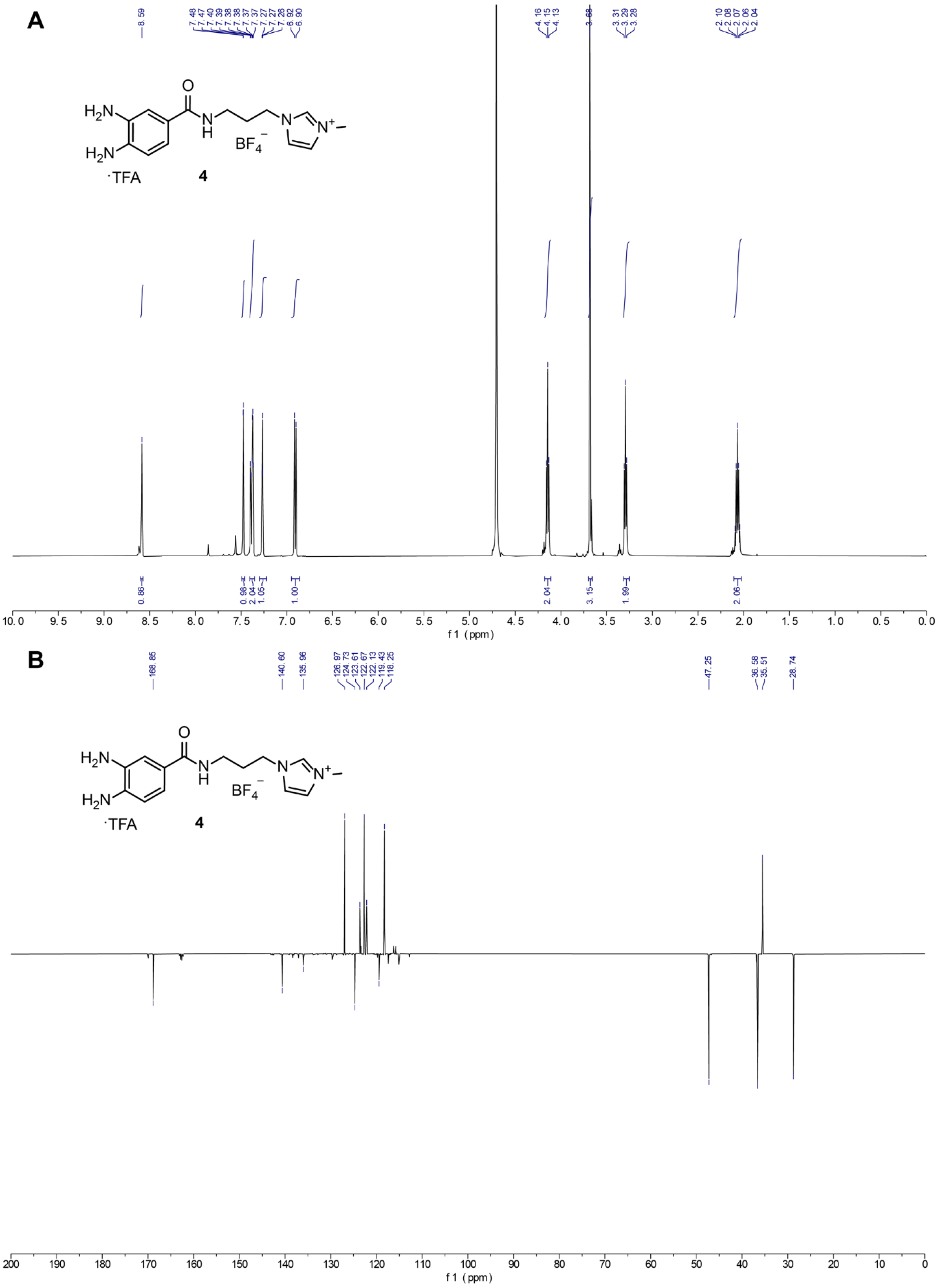

Figure S11. ^{1}H NMR (**A**) and ^{13}C APT NMR (**B**) results of compound **4**.

4.5 NMR result of compound 10

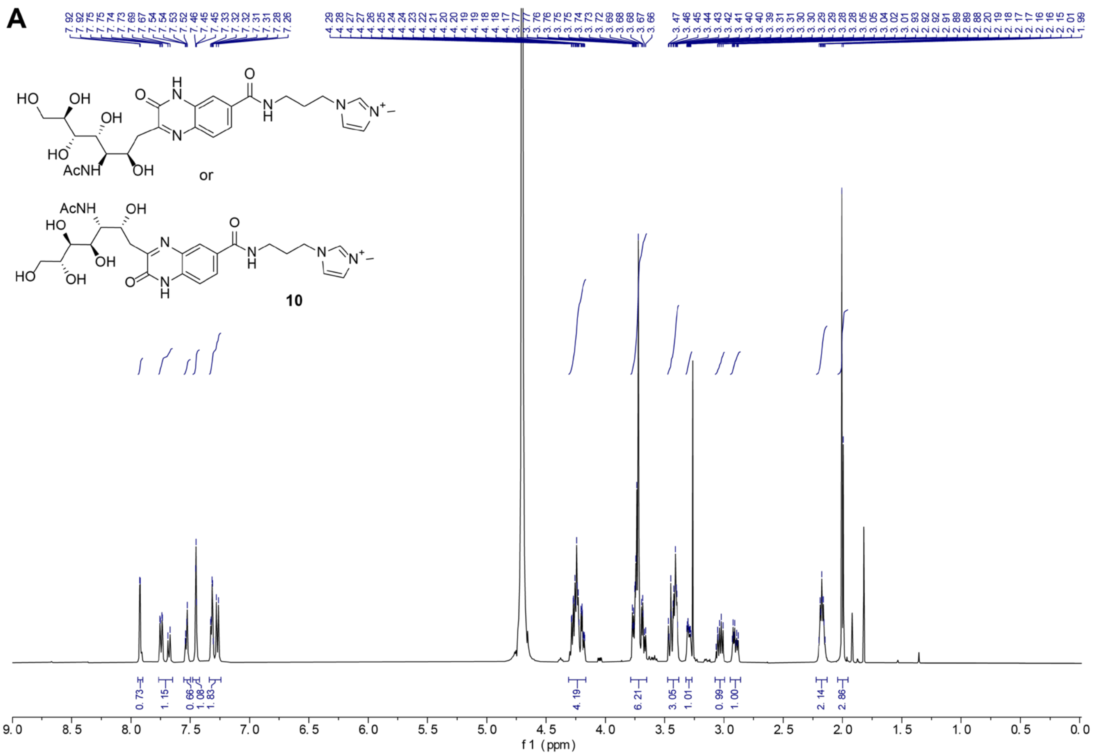

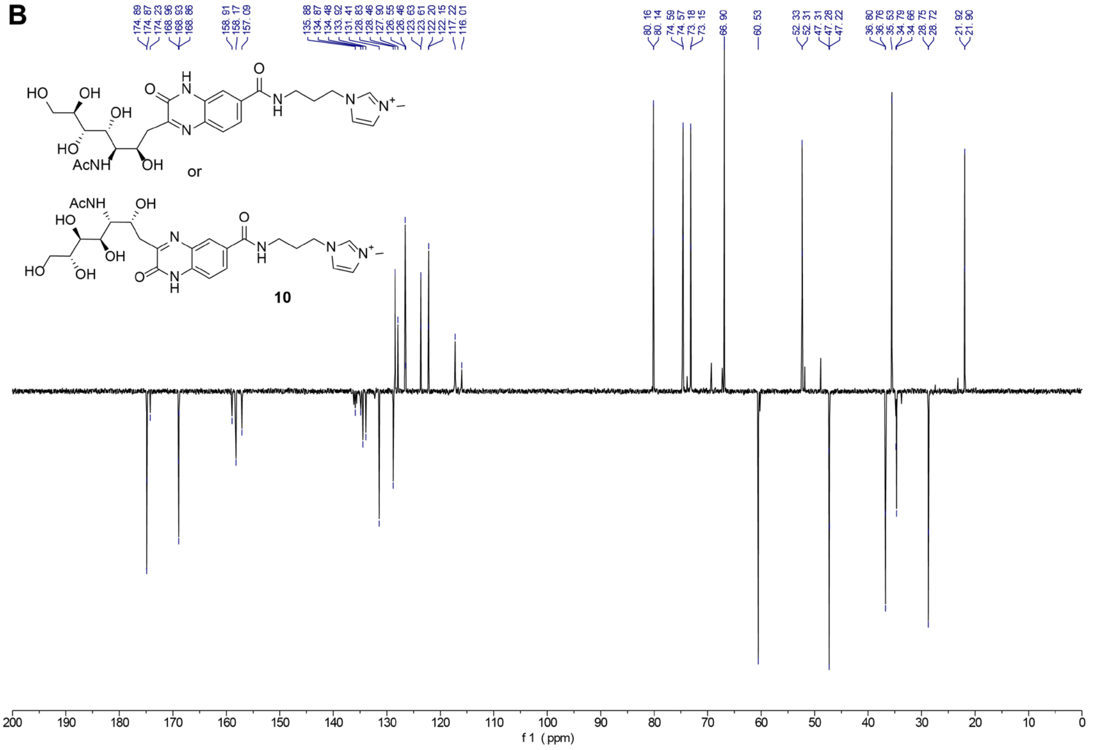

Figure S12. ^{1}H NMR (**A**) and ^{13}C APT NMR (**B**) results of compound **10**.
